# Modeling hepatic fibrosis in TP53 knockout iPSC‐derived human liver organoids

**DOI:** 10.1002/1878-0261.70119

**Published:** 2025-10-09

**Authors:** Mustafa Karabicici, Soheil Akbari, Ceyda Caliskan, Canan Celiker, Ozden Oz, Leman Binokay, Gökhan Karakulah, Serif Senturk, Esra Erdal

**Affiliations:** ^1^ Izmir Biomedicine and Genome Center (IBG) Dokuz Eylul University Health Campus Izmir Turkey; ^2^ Izmir International Biomedicine and Genome Institute (IBG‐Izmir) Dokuz Eylul University Izmir Turkey; ^3^ Department of Pathology Izmir Bozyaka Education and Research Hospital, University of Health Sciences Turkey; ^4^ Department of Medical Biology and Genetics, Faculty of Medicine Dokuz Eylul University Izmir Turkey; ^5^ Present address: Board of Governors Regenerative Medicine Institute Cedars‐Sinai Medical Center 8700 Beverly Blvd Los Angeles 90048 CA USA; ^6^ Present address: School of Biosciences University of Sheffield Sheffield UK; ^7^ Present address: Department of Histology and Embryology Faculty of Medicine, Masaryk University Brno Czech Republic

**Keywords:** CRISPR/Cas9, extracellular matrix remodeling, iPSC‐derived organoids, liver fibrosis, TP53 knockout

## Abstract

Hepatic fibrogenesis is characterized by the excessive accumulation of extracellular matrix proteins, ultimately predisposing to hepatocarcinogenesis. The lack of reliable models that faithfully recapitulate early stage fibrogenesis is one of the main limitations in identifying translationally relevant therapeutics. Here, we establish a model using CRISPR/Cas9‐mediated TP53 knockout iPSC (endoderm)‐derived human hepatic organoids (eHEPOs) to mimic human liver fibrosis. Transcriptomic profiling of TP53KO‐eHEPOs revealed enrichment of pathways associated with inflammation, ECM remodeling, and fibrosis, with notable alterations in pivotal fibrotic regulators. We also find increased expression of myofibroblasts and fibrosis markers (PDGFRB, COL1A1, COL3A1, COL11A1) and early liver cancer markers (GPC3 and MUC1). Histological analysis confirmed advanced fibrotic hallmarks and exposure to an exogenous profibrotic environment (pf‐ME) further enhanced these fibrotic phenotypes. This model provides a valuable platform for exploring the role of key driver genes, such as TP53, in the initiation and progression of fibrosis, enabling the study of hepatic progenitor cell transformation across diverse microenvironmental contexts. As such, it holds the potential for advancing early stage drug discovery and the identification of novel therapeutic targets for the treatment of liver fibrosis.

## Graphical abstract

This study developed iPSC‐derived human liver organoids with TP53 gene knockout to model human liver fibrosis. These organoids showed elevated myofibroblast activation, early disease markers, and advanced fibrotic hallmarks. The use of profibrotic differentiation medium further amplified the fibrotic signature seen in the organoids. Therefore, this model offers a promising platform for discovering antifibrotic therapies.

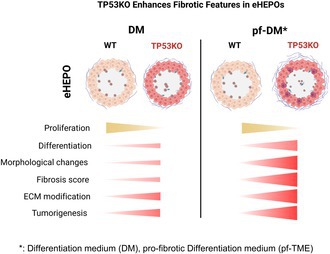

**Abbreviations**
AAV: adeno‐associated virus
ATM: ataxia telangiectasia mutated
CBh: adenovirus‐associated hybrid promoter
DM: differentiation medium
ECM: extracellular matrix
eHEPOs: iPSC (endoderm)‐derived human hepatic organoids
EM: expansion medium
EMT: epithelial‐mesenchymal transition
FDR: false discovery rate
FFA: free fatty acids
GFP: green fluorescent protein
GPC3: glypican‐3
gRNAs: guide RNAs
GSEA: gene set enrichment analysis
HCC: hepatocellular carcinoma
HSCs: hepatic stellate cells
IHC: immunohistochemical staining
iPSCs: induced pluripotent stem cells
MDC1: DNA damage checkpoint protein 1
MSigDB: molecular signatures database
MUC1: Mucin1
PAS: periodic acid‐Schiff stain
pf‐DM: profibrotic differentiation medium
pf‐ME: profibrotic environment
PMA: Phorbol 12‐myristate 13‐acetate
qPCR: quantitative polymerase chain reaction
RNA‐seq: RNA sequencing
TAA: thioacetamide
TMM: trimmed mean of M‐values
TP53BP1: TP53‐binding protein 1
WT: wild‐type

## Introduction

Liver fibrosis, a hallmark of chronic liver diseases and cancer initiation, is triggered by acute liver damage and characterized by an excessive amount of extracellular matrix protein accumulation. Current drug candidates focuson late‐stage related alterations in liver disease, and their effectiveness against liver fibrosis is limited. One main bottleneck is the limited translational relevance of mouse models to accurately mimic human physiology [1]. Additionally, there is a lack of alternative *in vitro* models that can more accurately recapitulate liver fibrosis and faithfully represent human physiological processes [2]. In this regard, iPSC‐derived hepatic organoids that contain various cell types, including kupffer, endothelial, and stellate cells, hold significant promise. Importantly, stellate cells, which are the main drivers of liver fibrosis, are crucial for representing more realistic drug responses for liver fibrosis [3]. A recent study by our group demonstrated that our iPSC‐derived eHEPO model faithfully replicates human liver physiology [4] and contains cells of both endodermal origin (ALB+) and mesenchymal origin (SMA_PDGFRB+ and CD68+), including hepatocytes, cholangiocytes, stellate cells, and myeloid line.

**Fig. 1:**
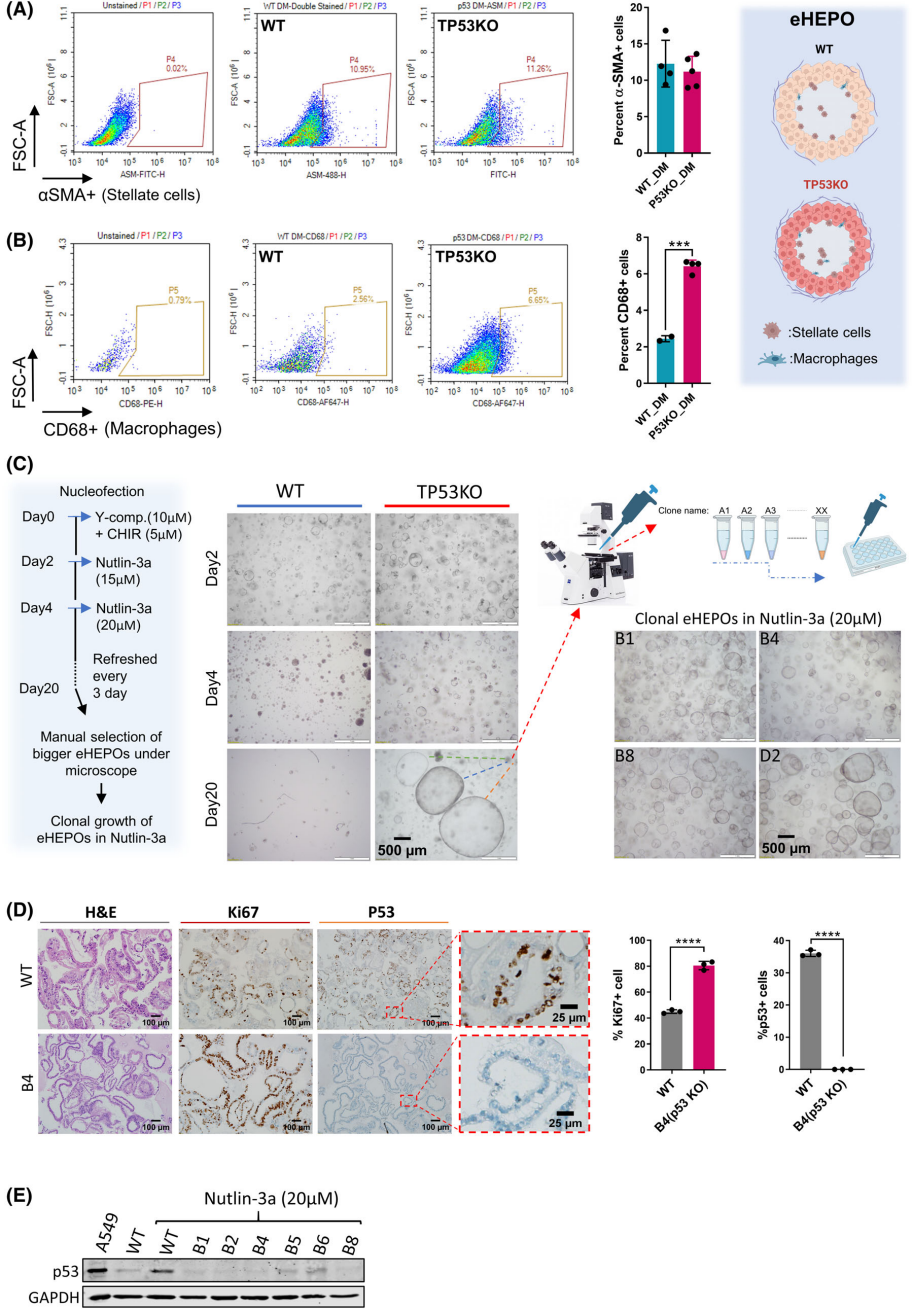
Generation of TP53KO‐eHEPOs through CRISPR/Cas9‐mediated engineering. (A) The percentage of a‐SMA+ stellate cells within DM medium (*n* = 3, SD). Cells were analyzed using the Novo‐Express cytometer, and results were analyzed using Novo‐Express flow cytometry software (version 1.6.2) (Santa Clara, CA, United States). (B) The percentage of CD68+ macrophage cells within DM medium (Student *t*‐test, *n* = 3). Cells were analyzed using the Novo‐Express cytometer, and results were analyzed using Novo‐Express flow cytometry software (version 1.6.2) (Santa Clara). Right panel showed a schematic presentation of eHEPO with stellate and macrophage cells. (C) The schematic depicts the optimization steps for the selection of TP53KO‐eHEPOs using Nutlin‐3a (20 μm) (left). The WT and TP53KO‐eHEPOs under Nutlin‐3a (middle) and clonal expansion of selected eHEPOs (right). (D) The IHC staining of B4 confirmed p53 KO status, while Ki67‐positive cell was significantly augmented in B4 (Student *t*‐test, *n* = 3, SD) (Scales: 100 and 25 μm) (‘> 0.05’ (n.s.), ‘≤ 0.05’ (*), ‘≤ 0.01’ (**),‘≤ 0.001’ (***), ‘≤ 0.0001’ (****)). (E) Western blot assay was performed after Nutlin‐3a treatment at 20 μm to verify P53 modification in TP53KO clones. GAPDH is used as a loading control (*n* = 3).

Furthermore, we confirm hepatic stellate cells and macrophages in our organoid model with a‐SMA and CD68 flow cytometry and staining (Fig. 1A,B and Fig. S1A,B). Therefore, we utilized this model to mimic hepatic fibrosis within the knockout condition of TP53, the main driver gene of liver fibrosis initiation. TP53 has at least two distinct roles in developing liver fibrosis. Firstly, as a tumor suppressor gene, among the most frequently mutated genes in tumor hepatocytes (35–50% of liver cancer cases), it safeguards normal cells from premalignant transformations [5]. In addition to this, p53 can suppress tumorigenesis non‐cell autonomously by promoting an antitumor microenvironment, such as downregulating the production of extracellular matrix (ECM) proteins, upregulating ECM‐degrading enzymes, and secreting a variety of immune modulators [6]. To this end, ablation of a p53‐dependent senescence program in hepatic stellate cells (HSCs) has been shown to increase liver fibrosis and enhance the transformation of adjacent hepatocytes into hepatocellular carcinoma (HCC). Mechanistically, the wild‐type (WT) p53 senescent stellate cells canonically promote tumor‐inhibiting M1 state macrophage polarization, which attacks and clears senescent cells to resolve fibrotic tissue formation. Conversely, p53‐deficient stellate cells skew cells to a tumor‐promoting M2 state and support the malignant transformation of adjacent precursor cells. These tumor‐promoting macrophages play a pivotal role in inducing the transformation of stellate cells into activated myofibroblasts through TGF‐β‐like stimulations. These activated stellate cells, in turn, secrete various factors into the tumor microenvironment, thereby fueling a feedforward loop that aids in the maintenance of these tumor‐promoting macrophages [7, 8]. The secretome of these cells modifies the microenvironment and orchestrates the progression of liver progenitor and/or stem cells toward malignant transformations and cancer initiation. Due to the interplay between these myofibroblasts and macrophages, which remodel the ECM and the immune microenvironment in the fibrotic liver, we collected their secretomes, also called profibrotic microenvironment (pf‐ME) or profibrotic differentiation medium (pf‐DM) when combined with differentiation medium (DM), and cultured them with TP53KO‐eHEPOs to further recapitulate liver fibrosis initiation and progression in our model.

In this model, we observed that altered, fibrosis‐related pathological changes include atypical cells, pseudo‐glandular‐tubular rosettes, steatohepatitis‐like inflammatory areas, and ballooning‐like hepatocytes. In accordance, we confirmed an increased accumulation of collagen and related gene expressions, including *COL1A1*, *COL3A1*, *COL11A1*, and *COL24A1*. More importantly, the expression of myofibroblast and fibrosis marker *PDGFRB*, and *IL‐33* is accompanied by a paralleled increase of early liver disease markers GPC3 and MUC1 in TP53KO‐eHEPOs [3, 9, 10, 11, 12, 13]. Finally, Wnt/TGF‐β signaling alterations and other fibrosis‐related pathways correlated with increased fibrosis in TP53KO‐eHEPOs. Hence, this model offers a valuable tool for investigating the impact of early driver genes, such as TP53, on disease initiation. It can be used to study hepatic progenitor cell transformation in diverse microenvironments, potentially providing a platform for early‐stage drug development and identification of novel candidates for liver fibrosis treatment.

## Methods

### Organoid culture

The generation and maintenance of the organoids were carried out as previously described [4]. The organoids, which were previously produced from iPSCs and preserved in the liquid nitrogen, were thawed. iPSC production and permissions were described in a previous study, and mycoplasma contamination was routinely checked in iPSCs and organoid stages. The pellet was then mixed with 60% matrigel (Corning, Arizona, USA) and 40% medium (AdDMEMF12) and seeded on plates as 30–50 μL droplets (6‐wp, 8–10 drops). The expansion medium (EM) prepared in AdDMEMF12 (Gibco, New York, USA) with 100 ng·mL^−1^ fibroblast growth factor 10 (FGF10), 25 ng·mL^−1^ hepatocyte growth factor (HGF), 10 mm nicotinamide, 5 μm A83.01, 10 μm forskolin, 1% B27, 1.25 mm N‐acetylcysteine, 10 nm gastrin, 50 ng·mL^−1^ epidermal growth factor (EGF), and 10% R‐spo1 CM (in‐house). To differentiate immature hepatocytes into functional hepatocytes, organoids were kept in AdDMEMF12 with 1% B27, EGF (50 ng·mL^−1^), gastrin (10 nm), HGF (25 ng·mL^−1^), FGF19 (100 ng·mL^−1^), A8301 (500 nm), DAPT (10 μm), BMP7 (25 ng·mL^−1^), and dexamethasone (30 μm) including differentiation medium (DM). Functional experiments were performed on early passage organoids (passages 3–6) and kept in DM medium for 12 days.

### TP53 gRNA

The gRNA used for TP53 knockout was designed and transformed into the Addgene Px458‐GFP vector (pSpCas9(BB)‐2A‐GFP (PX458), Plasmid #48138), which contains an adenovirus‐associated hybrid promoter (CBh) as previously reported [14]. Accordingly, the gRNA sequence targeting exon 4 is TP53‐F5′ CACCgCCATTGTTCAATATCGTCCG‐3′, TP53‐R5′‐AAACCGGACGATATTGAACAATGGc‐3′. This design facilitates a homozygous frameshift mutation in the 4th exon region of TP53, in which a single adenine nucleotide is added after 3 nucleotides following the PAM sequence (GGG).

### Western blot

Organoids were collected and removed from the matrigel by washing with cold PBS containing protease and phosphatase inhibitors (6wp, 8–10 dots/well, 50 μL/dome). RIPA buffer (50 mm Tris/HCl, pH 8.0, 150 mm NaCl, 1% NP‐40, 0.5% sodium deoxycholate, 0.1% SDS, protease inhibitors) was added to the organoid pellet and kept on ice for 30 min (Vortex every 5 min). As a final step, after 30 s of sonication, the cell pellet was precipitated at 13,200 ***g*** at +4 °C for 20 min. and the supernatant was collected. BCA assay (Thermo, Rockford, IL, USA) was used to determine the protein concentration (60 μg protein loaded). The TP53 (DO‐7) Mouse mAb (#48818) and GAPDH (sc‐25 778) antibodies were used at 1 : 1000 concentration. Blots were imaged on Licor Odyssey (Lincoln, NE, U SA).

### Reporter assays

TCF/LEF and pSBE4luc/pRL‐TK luciferase methods were performed as described before [15]. Before the experiment, organoids were collected with cold AdDMEM‐F12 and trypsinized into a single cell. For each experimental condition, 100 ng of TCF/LEF, 40 ng of Renilla, 200 ng of pSBE4luc, and 30 ng of pRL‐TK plasmids were transfected 1 : 3 using Lipofectamine™ 3000 transfection reagent (Thermo Scientific, CA, USA). Luminometric measurements were taken 24 h after transfection (LB960; Berthold, Tennessee, USA). Analyses were performed with graphpad Prism.

### Nucleofection

Nucleofection was performed using the Amaxa P3 Primary Cell 4D‐Nucleofector™ X Kit's EX147 or DS150 programs according to the manufacturer's instructions, with optimization and modifications. The nucleofection protocol was applied to healthy WT organoids to obtain TP53KO‐eHEPOs. Briefly, Y‐27632 (Tocris, Minneapolis, MN, USA) (10 μm) and CHIR 99021 (Tocris) (10 μm) were added to the cell medium 2 days before the transfection of early passage WT organoids. After 24 h, this medium was withdrawn and replaced with a medium containing Y‐27632 + CHIR 99021 + 1.25% DMSO. After the overnight incubation, the cells were incubated with trypsin for 7–10 min. For transfection, 5 μg gRNA plasmid and 100 μL transfection reagent were used, and a total of 5 × 10^5^ cells were transfected and incubated for 15 min at room temperature. After transfection, prewarmed (37 °C) 400 μL of Y‐27632 and CHIR 99021 containing organoid medium were added to the cells, and the cells were kept in an ultra‐low attachment 24‐well plate in the incubator for 4–6 h, and inoculated into matrigel (7 dots/7 × 10^4^ cells). Cells were visualized under a fluorescent microscope 48 h after transfection. Transfection efficiency was measured by flow cytometry.

### TP53K0‐eHEPO selection medium

To select and enrich TP53KO‐eHEPOs from transfected organoids, Nutlin‐3a was added to the EM medium. Nutlin‐3a stabilizes TP53 by interfering with its binding to MDM2, leading to p53‐mediated cell cycle arrest and apoptosis. However, cells with a knockout of TP53 can escape from this fate and grow as large organoids. To obtain pure selected clones after transfection, organoids were cultured for 20 days with a relatively high dose of Nutlin‐3a (20 μm). Excessively growing organoids were selected under the microscope using a 200 μL tip and separately seeded for clonal expansion and further confirmation of their KO status. After this confirmation step, we removed Nutlin‐3a from the TP53KO organoids before conducting any experiments, leveraging the long‐term stability of the adeno‐associated virus (AAV) vector system. To ensure the maintenance of the TP53KO status, we repeated Nutlin‐3a treatment before preparing frozen stocks (once every 3–6 months, depending on usage of stocks) but did not apply it after thawing or before starting any experiments.

### The preparation of profibrotic DM (pf‐DM) medium

To prepare the pf‐DM medium, we combined activated LX2 stellate cells (myofibroblast) and M2‐polarized macrophage secretomes. LX2 stellate cells were treated with TGFβ‐1 (10 ng·mL^−1^) for 24 h to induce myofibroblast formation. The medium was replaced with fresh medium, and the secretome was collected 48 h later [16]. To confirm their transformation, we assessed the expression levels of the myofibroblast markers *PDGFRβ* and *COL1A1* and observed morphological changes (Fig. S2A). To generate M2‐type macrophages, THP‐1 monocytic cells were treated with 200 ng·mL^−1^ phorbol 12‐myristate 13‐acetate (PMA) for 48 h to differentiate them into macrophages. To allow the cells to recover, the medium was refreshed, and they were left without further treatment for 2 days. They were then induced with IL‐4 (25 ng·mL^−1^) for protumorigenic M2 polarization for 24 h. After incubation, the medium was refreshed, and the secretome was collected 48 h later [17, 18]. To confirm their polarization to M2, we assessed the expression levels of M2 macrophage‐specific markers, including *CCL22*, *CD206* (M2 marker), and *MCP1* (M1 marker), and observed associated morphological changes (Fig. S2B). Subsequently, both secretomes (also called pf‐ME) were collected, filtered, and added to WT and TP53KO‐eHEPOs in equal proportions, accounting for 15% each, to create a total of 30% profibrotic differentiation medium (pf‐DM), was used to promote hepatic fibrogenesis.

### Real‐time qPCR

Total RNA was isolated from organoids using the GeneJET RNA purification kit (Cat #K0732; Thermo Fisher Scientific) and converted to cDNA using a Maxima First Strand cDNA Synthesis kit (Cat #K1642; Thermo Fisher Scientific). The expression levels were determined in quadruplicate on a 7500 Fast RT PCR System (Applied Biosystems, CA, USA) using the TaqMan™ Universal Master Mix or Promega GoTaq. The primers are given in Table S1.

### Flow cytometry

For all experimental conditions, organoids were collected with ice‐cold AdDMEMF12 and centrifuged at 400 ***g*** for 5 min at 4 °C to remove matrigel. Cells were trypsinized for 10 min at 37 °C to obtain single‐cell suspensions. The single cells were washed with 1× FACS buffer (2% FBS, 0.5% EDTA, 2.5% HEPES) and analyzed using a BD LSR Fortessa flow cytometer (Becton Dickinson, Heidelberg, Germany). Data were processed with flowjo software as previously described [15].

### Albumin ELISA

The Albumin ELISA assay was performed according to the manufacturer's protocol. Briefly, 24 h after the last media refreshment, organoid culture media were collected, and the concentration of albumin was measured using a Human Albumin ELISA Kit (Bethyl, TX, USA). The concentration of albumin was calculated as ngALB/day/10^6^ cells [15].

### Immunohistochemical staining (IHC)

All organoid samples were fixed with formalin and embedded in paraffin. Before staining, sections were deparaffinized and dehydrated using a graded ethanol series using routine protocols. After dehydration, samples were stained with primary antibodies or indicated dyes. Slides were examined with an Olympus BX61 microscope (Olympus Corporation, Tokyo, Japan) according to the positivity rate method.

For OCT staining, on day 12, differentiated organoids were collected without fragmentation and centrifuged at 200 ***g*** for 5 min, and the matrigel was removed. The organoids were fixed overnight in 10% formalin buffer at 4 °C followed by incubation in 30% sucrose for 2 or 3 days and embedded in OCT. The OCT blocks were then sectioned at 8 μm thickness, transferred to slides, and left for about 1 h to dry at RT. OCT was removed by immersing it in distilled water for 5 min. Then, they were incubated with 0.5% permeabilization buffer (15 min) and blocking buffer (1% BSA, 10% goat serum, 0.1% Tween 20, 0.3 M glycine for 10 mL) (1 h). After blocking, the samples were incubated overnight with primary antibodies in the same blocking buffer. The antibodies used in the study are as in Table S2. The images were taken with the C‐Apochromat 40×/1.2 W Korr FCS M27 lens on the LSM 880 Axio Observer confocal device and edited with the imagej program (Version 1.54f).

### Preparation of RNA‐seq samples

WT and TP53KO organoids were collected on day 12 after differentiation (in the DM medium). RNA was isolated by RNeasy Plus Mini Kit (Cat no: 74134). For the sequencing, 1.5 μg total RNA was used for the construction of the sequencing libraries. RNA quality control was performed by Qubit 3 fluorometer (ABD, #Q33216; Thermo Fisher, Eugene, OR, USA) and 2100 Bioanalyzer (ABD, #G2939BA; Agilent, Santa Clara, CA, USA). RNA libraries for RNA‐seq were prepared using the Illumina Stranded mRNA Prep kit (ABD, #20040534; Illumina). For sequencing, the Illumina NovaSeq 6000 platform was used to obtain an average of 150 bp 30 million paired‐end reads for each sample.

### Analysis of RNA‐seq data

The GRCh38 human reference genome, also known as hg38, and its associated reference annotation (version 42) in the Gene Transfer Format (GTF) were downloaded from the official website of the GENCODE project, which can be accessed at https://www.gencodegenes.org/. To align the raw sequencing reads to the human reference genome, we utilized the R package Subread aligner (v2.0) [19] with the following command: subread‐align ‐t 0 ‐i {index} ‐T 16 ‐r {input 1.fastq.gz} ‐R {input 2.fastq.gz} ‐o {output.bam}. Subsequently, we employed the featureCounts function [20] from the R package to count the number of reads overlapping with GENCODE‐annotated genes. In this step, the following command was executed: featureCounts(files = {infile. bam}, annot.ext = “{infile. gtf}”, is GTF AnnotationFile = T, GTF.featureType = “exon”, GTF.attrType = “gene_id”, countMultiMappingReads = T, isPairedEnd = T). Fragments per kilobase million (FPKM) values were then computed for each gene across all samples. To enhance the detection sensitivity of differentially expressed gene features, we applied a filter to include genes with mean expression values greater than or equal to one FPKM in both the control and B4 (TP53KO) groups. The filtered count matrices were subsequently employed for the differential expression analysis to identify genes with varying expression levels between the control and B4 groups. This analysis was performed using the EdgeR package (v3.40.2) within the R environment. Trimmed mean of M‐values (TMM) normalization was applied to the count values, and dispersions were estimated using the estimateDisp function for each comparison. To calculate the false discovery rate (FDR) for each gene, we utilized the exactTest function of edgeR.

### Gene set enrichment analysis (GSEA) and visualization

Molecular Signatures Database (MSigDB) (v2023) and the GSEA (v4.3.2) software of the Broad Institute were used to identify enriched pathways (GO, KEGG, HALLMARKS, REACTOME, ONCOGEN) within the expressed gene list. To visualize significantly differentially expressed genes, we employed volcano plots, heatmaps, and dot plots. The complex heatmap package (v2.15.04) in R was used to create these heatmaps, and the ‘ggplot2’ package (v3.4.2) in R was used to create volcano plot and dot plots, enabling the clustering and visualization of gene expression patterns across samples. All statistical analyses were conducted using R 4.2.2 (https://www.r‐project.org/). We considered all probability values statistically significant at *P* < 0.05.

### Statistical analysis

Statistical analyses were done using graphpad Prism 8 (graphpad Software, Inc., California, United States) software for bar graphs in Figs 1D,E, 3A, 4A–D and 5B,C. A two‐tailed unpaired Student's *t*‐test was used to determine statistical significance between the two experimental groups within a 95% confidence interval, if otherwise mentioned in the figure legend. Differences between groups were considered as ‘> 0.05’ (n.s.), ‘≤ 0.05’ (*), ‘≤ 0.01’ (**),‘≤ 0.001’ (***), ‘≤ 0.0001’ (****). All data represented as mean standard deviation (SD) of at least three (*n* ≥ 3) independent experiments. In the transcriptomic analysis, all data represent statistically significant at *P* ≤ 0.05 value.

## Results

### Optimization and validation of a TP53KO‐driven organoid model

To model and elucidate the mechanisms underlying the initiation of liver fibrosis, we first mimic loss‐of‐function of the p53 gene in healthy human iPSC‐derived liver organoids by CRISPR/Cas9 gene editing. To achieve this, we used predetermined guide RNAs (gRNAs) designed to introduce a homozygous frameshift mutation in exon 4 of the *TP53* gene (Fig. S2C). This gRNA shows a single‐nucleotide adenine insertion after three nucleotides from the PAM sequence (Fig. S1C). Following several protocol optimizations, we achieved a 70–80% transfection efficiency using nucleofector gRNA transfection (Fig. S1D). Cell viability and transfection ratio were measured by flow cytometry 2–3 days post‐transfection. In cases where the transfection efficiency falls below 50%, GFP‐positive cells can be enriched by cell sorting for further analysis (Fig. S3A). After nucleofection, we used MDM2‐p53 interaction inhibitor Nutlin‐3a to selectively enrich TP53KO‐eHEPOs (Fig. 1C left panel). We observed that cell death occurred in the control (empty plasmid) transfected WT‐eHEPO group after 3–4 days of treatment with Nutlin‐3a (Fig. 1C middle panel). Specifically, certain cells within the TP53KO plasmid‐transfected group expanded and grew in the Nutlin‐3a‐treated condition, indicating that the CRISPR/Cas9 system successfully edited the TP53 gene [21, 22, 23]. These large organoids in the TP53KO group were harvested using a 200 μL pipette tip and then cultured for clonal growth (Fig. 1C right panel). To determine P53 protein expression within these clones, we followed a comprehensive multi‐step validation strategy that involved Sanger sequencing (Fig. 1A and Fig. S3B) followed by IHC staining (Fig. 1D), and western blot analysis (Fig. 1E). Firstly, the results of Sanger sequencing consistently indicated the presence of a single‐nucleotide adenine insertion in all clones (Fig. 1A and Fig. S3B). Additionally, western blot analysis demonstrated a notable ablation in P53 protein levels, particularly within the B2, B4, and B8 clones. This observation was further confirmed by IHC staining, which confirmed the lack of P53 protein expression in the B2, B4, and B8 clones (Fig. S3C). Based on the significant increase in proliferation capacity (Ki67 ratio), along with the distinct loss of P53 staining patterns observed, we selected clone B4 for further biological and transcriptomic analysis (Fig. 1D and Fig. S3C). Taken together, we established a CRISPR/Cas9‐mediated TP53KO liver organoid model, validated through multi‐level analyses, and clone B4 was selected for further analysis due to its high proliferation and P53 deficiency.

### TP53KO‐eHEPOs represent versatile proliferation and differentiation properties depending on the medium‐derived stimulation

**Fig. 2:**
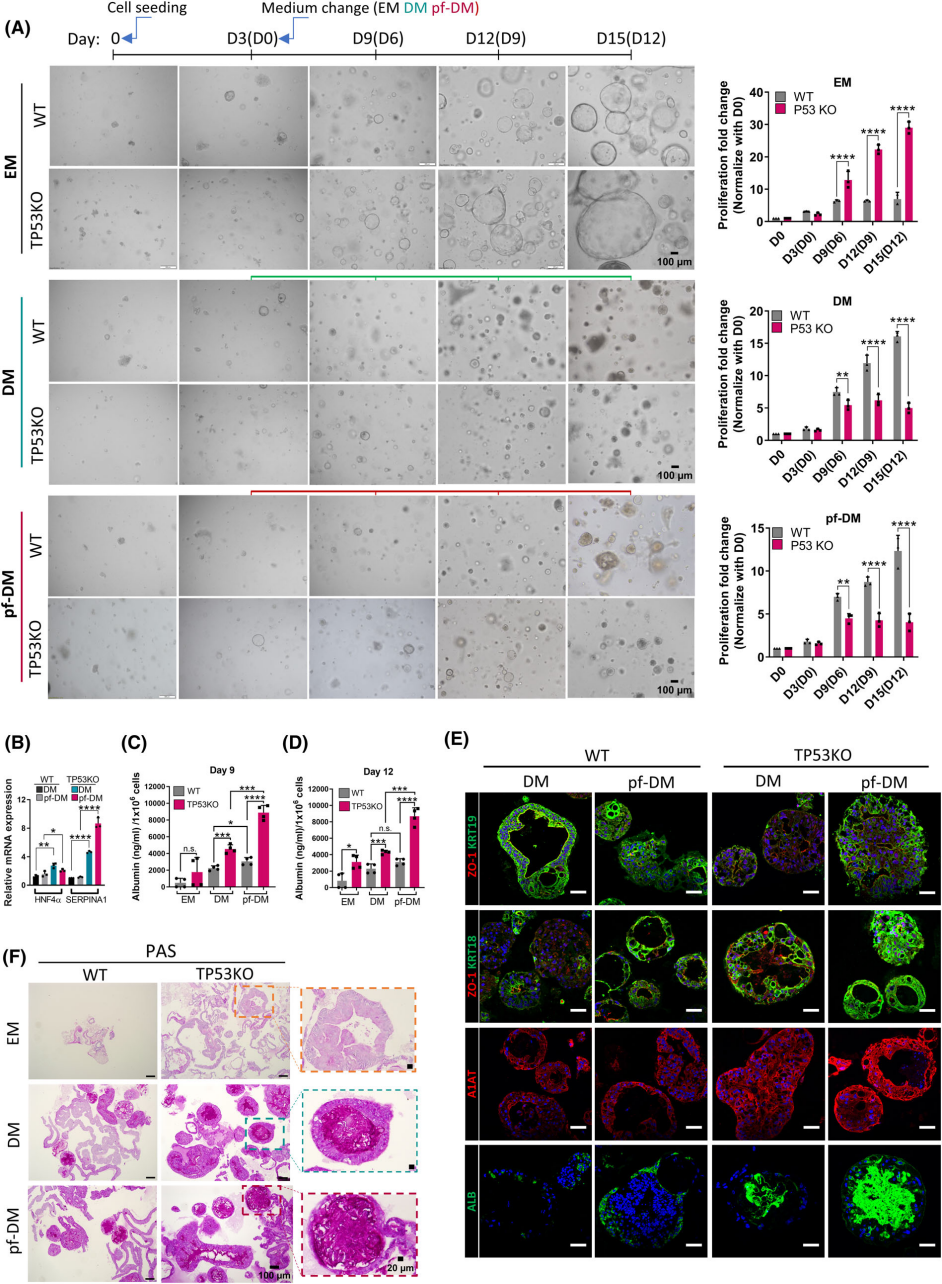
TP53KO‐eHEPOs represent augmented proliferation and differentiation properties depending on the medium‐derived stimulation. (A) The proliferation of WT and TP53KO‐eHEPOs in different medium conditions. All organoids were seeded at day 0 and three days later (day 3) one group was held in EM medium, while others were replaced with DM and pf‐DM mediums (Scales: 200 μm (10×)), (left). At each time point, organoids were lysed with Promega's Cell‐Titer‐Glo® 3D reagent, and the accumulated signal was collected. The graphs were prepared in graphpad Prism. (‘> 0.05’ (n.s.), ‘≤ 0.05’ (*), ‘≤ 0.01’ (**), ‘≤ 0.001’ (***), ‘≤ 0.0001’ (****), (Student *t*‐test, *n* = 3, SD)) (right). (B) The qPCR of EM, DM, and pf‐DM‐treated WT and TP53KO‐eHEPOs for differentiation markers. (‘> 0.05’ (n.s.), ‘≤ 0.05’ (*), ‘≤ 0.01’ (**), ‘≤ 0.001’ (***), ‘≤ 0.0001’ (****), (Student *t*‐test, *n* = 3, SD)). (C) The eHEPO secretome was collected on day 9 after DM or pf‐DM induction and albumin ELISA was performed. The results were normalized based on the eHEPO cell count, which was determined after the trypsin‐mediated single‐cell isolation process. Graphs prepared in graphpad Prism (‘> 0.05’ (n.s.), ‘≤ 0.05’ (*), ‘≤ 0.01’ (**), ‘≤ 0.001’ (***), ‘≤ 0.0001’ (****), (Student *t*‐test, *n* = 4, SD)). (D) Albumin ELISA at day 12 (*n* = 4, SD). (E) IF staining of WT and TP53KO‐eHEPOs for differentiation markers from OCT sections (Scale: 50 μm), (*n* = 3). (F) Periodic acid‐Schiff stain (PAS) performed in the WT and TP53KO‐eHEPOs to better understand well‐differentiated cell markers for hepatocytes represent elevated signal of TP53KO‐eHEPOs (Scale: 100 μm, zoom in scale: 20 μm), (*n* = 3).

The *TP53* gene is a well‐known guardian of cells, protecting against the accumulation of mutations and unwanted cell state differentiation, and maintaining a balance between proliferation and differentiation [24, 25]. To evaluate the proliferative capacity of cells, we used Ki67, which is commonly used as a proliferation marker [26]. In our study, we observed that knockout of TP53 in the eHEPOs led to increased Ki67 staining in the expansion medium (EM), indicating heightened cell proliferation (Fig. 1D). However, when the cells are induced to differentiate with the differentiation medium (DM), there is a noticeable shift toward differentiation (Fig. 2A and Fig. S4A). Additionally, our qPCR results confirmed a significant augmentation in the expression of well‐differentiation‐related genes of hepatocytes including *HNF4a* and *SERPINA1* (also known as *A1AT*) in TP53KO‐eHEPOs in DM medium (Fig. 2B). Furthermore, we observed that TP53KO‐eHEPOs exhibited increased albumin secretion into the medium at days 9 and 12 in the DM medium compared to WT. Interestingly, the profibrotic microenvironment (pf‐ME) further enhanced this effect in TP53KO‐eHEPOs, while it had no significant effect on the WT counterparts at day 12 (Fig. 2C,D). In addition to these results, we examined several liver‐specific markers using IF staining. Initially, we observed that ALB staining was significantly enriched in TP53KO‐eHEPOs, with the staining intensifying in the profibrotic differentiation medium (pf‐DM). Additionally, the levels of alpha‐1‐antitrypsin (*A1AT*) in TP53KO organoids increased in both DM and pf‐DM. Furthermore, we investigated cytokeratin expression in our eHEPO model, specifically focusing on the hepatocyte marker KRT18 and the cholangiocyte and hepatoblast marker KRT19 [27] (Fig. 2E). Our staining revealed increased KRT18 expression in TP53KO‐eHEPOs under both DM and pf‐DM conditions. Conversely, KRT19 expression decreased in TP53KO‐eHEPOs grown in DM medium compared to WT‐eHEPOs. Interestingly, KRT19 expression in TP53KO‐eHEPOs restores to levels comparable to WT‐eHEPOs under pf‐DM conditions, indicating that the induction of KRT19 by pf‐DM occurs independently of TP53. This suggests that TP53 does not directly control upregulation of KRT19, and likely other factors drive this response (Fig. 2E). Additionally, the PAS assay, a recognized method for evaluating hepatocyte function for glycogen storage, provided further confirmation that TP53KO‐eHEPOs display well‐differentiated hepatocyte‐like functions [28] (Fig. 2F). Furthermore, we also investigated ALB, A1AT, and PAS staining in our other B2 and B8 TP53KO‐eHEPOs and observed significantly increased in TP53KO clones compared to WT (Fig. S4B). In summary, these experiments revealed that the loss of the *TP53* gene leads to enhanced proliferation in the undifferentiated state while promoting hepatic functions with altered histopathological changes in DM and pf‐DM medium.

### Transcriptome signature of TP53KO‐eHEPOs reveals the fibrotic transformation

**Fig. 3:**
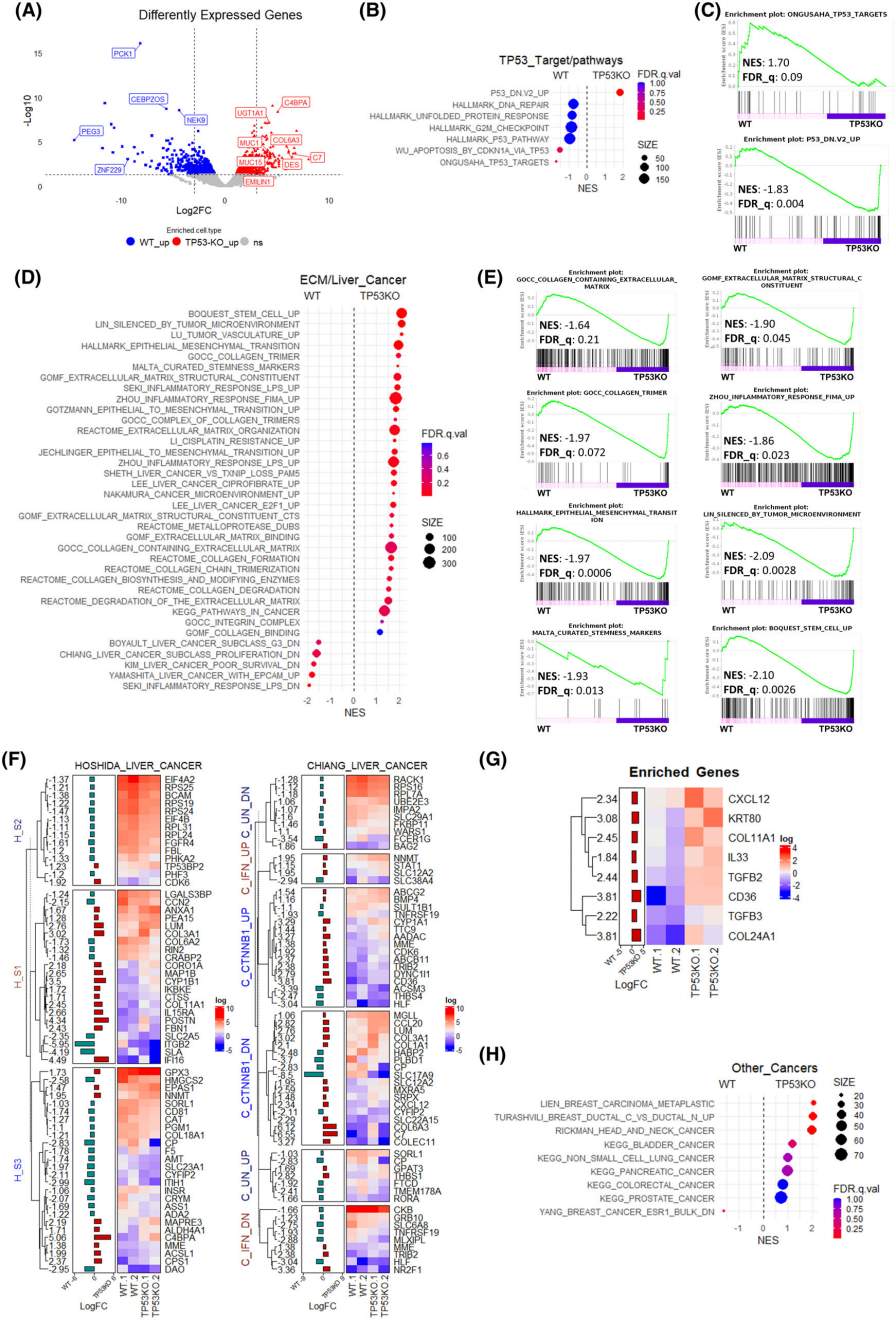
Transcriptomic Profile of P53KO‐eHEPOs. (A) Volcano plot of differentially expressed genes (DEG) in WT and TP53KO‐eHEPOs (up: 624; down: 711 genes). Hepatocyte function‐related genes (such as PCK1, PEG3) enriched in WT, while ECM modification and early liver disease‐related genes (such as; COL6A1, DES, and MUC1) enriched in TP53KO‐eHEPOs. (B) The dot plot of selected TP53‐related signal pathways from gene set enrichment analysis (GSEA). The figure represents the minus multiplication value of the normalized enrichment score (NES score). Low FDR. *q* values represent a more enriched signal pathway score (FDR. *q* < 0.25). (C) The gene set enrichment analysis plot of TP53 targets represents a positive correlation with WT, while downregulated TP53‐related scores are positively correlated with TP53KO‐eHEPOs. (D) The dot plot of selected ECM, Inflammation, and ECM‐related signal pathways enrichment score. The figure represents the minus multiplication value of the NES score. (E) The GSEA plot of ECM, collagen trimers, inflammation, EMT, tumor microenvironment, and Stemness scores represents a positive correlation with TP53KO‐eHEPOs. (F) The heatmap of Chiang and Hoshida's well‐characterized liver cancer‐related subsets in WT and TP53KO‐eHEPOs. The plots represent only statistically significant genes at *P* ≤ 0.05 value in our data set. The expression values represented in the matrix normalized with log transformation and default LogFC values were added as an annotation. (G) The heatmap of fibrosis, inflammation, and ECM modification related enriched genes in the TP53KO‐eHEPO (log(matrix values)), default (logFC), *P* ≤ 0.05. (H) The dot plot of enrichment scores of other cancers in TP53KO‐eHEPOs (*P* ≤ 0.05).

To explore the transcriptomic changes associated with TP53 and its potential effect on the fibrotic transformation of mature hepatocytes, we performed RNA‐seq analysis on WT and TP53KO organoids in DM conditions (Fig. S5A). The analysis revealed that 623 genes were upregulated and 710 genes were downregulated significantly in TP53KO‐eHEPOs (Fig. 3A) (Table S3). To better characterize these results, we first examined the transcriptional changes of TP53 target genes. The TP53KO‐eHEPOs had an attenuated expression pattern compared to the WT group. Furthermore, GSEA plots indicated that the ‘ONGUSAHA_TP53_TARGETS’ score, as well as scores related to P53 functions, were enriched in the WT group. In contrast, the TP53KO‐down regulation‐related score, P53.DN.V2.UP, was upregulated in TP53KO‐eHEPOs (Fig. S3B,C).

To explore further transcriptomic changes enriched in either WT or TP53KO‐eHEPOs, we conducted additional analyses across multiple collections, including GOCC, GOMF, GOBP, HALLMARKS, KEGG, ONCOGENE, and REACTOME (Fig. S5B–E and Table S4). This analysis showed that several signaling pathways associated with hepatic fibrosis were enriched in TP53KO‐eHEPOs. These pathways included ECM modification, collagen signaling, inflammatory response, epithelial–mesenchymal transition (EMT), tumor microenvironment, and stemness (Fig. 3D,E). We also confirmed that TP53KO‐eHEPOs had an enriched correlation score with HOSHIDA's liver subclass S1, which is related to changes in the Wnt/β‐catenin signaling pathway. TP53KO‐eHEPOs exhibit an enriched score for interferon signaling related to the CHIANG liver cancer subclass (Fig. 3F). Additionally, we observed a relatively higher expression of YAMASHITA's EPCAM_UP cluster in our WT‐eHEPOs, indicating a possible decreased Wnt signaling activity in TP53KO‐eHEPOs (Fig. S5D). Furthermore, we observed upregulation of several gene scores related to fibrosis, inflammation, and ECM modification including *COL11A1, COL24A1, TGFB2, TGFB3, CD36, IL33, CXCL12*, and KRT80 [29, 30, 31] (Fig. 3G).

We also investigated alterations in TP53KO‐eHEPOs related to *TP53*'s well‐defined roles in the cell cycle, apoptosis, and metabolism. Supporting the well‐established role of *TP53* as a tumor suppressor in diverse cancers, our fibrotic TP53KO‐eHEPOs displayed a close correlation to cancer models that often harbor nonfunctional p53 due to truncated proteins or various mutations compared to WT‐eHEPOs (Fig. 3H). Furthermore, we observed that pathways associated with glycolysis, gluconeogenesis, and amino acid metabolism were enriched in WT‐eHEPOs. Conversely, drug/xenobiotic metabolism pathways were enriched in TP53KO‐eHEPOs (Fig. S5E). Consequently, transcriptomic analysis of TP53KO‐eHEPOs revealed significant alterations in gene expression related to fibrosis, inflammation, and ECM remodeling. Pathways associated with hepatic fibrosis, EMT, and the tumor microenvironment were significantly enriched in TP53KO‐eHEPOs. These findings showed that TP53KO‐eHEPOs reveal fibrotic transformation and related pathological changes.

### Fibrotic signature is significantly increased in TP53KO organoids

**Fig. 4:**
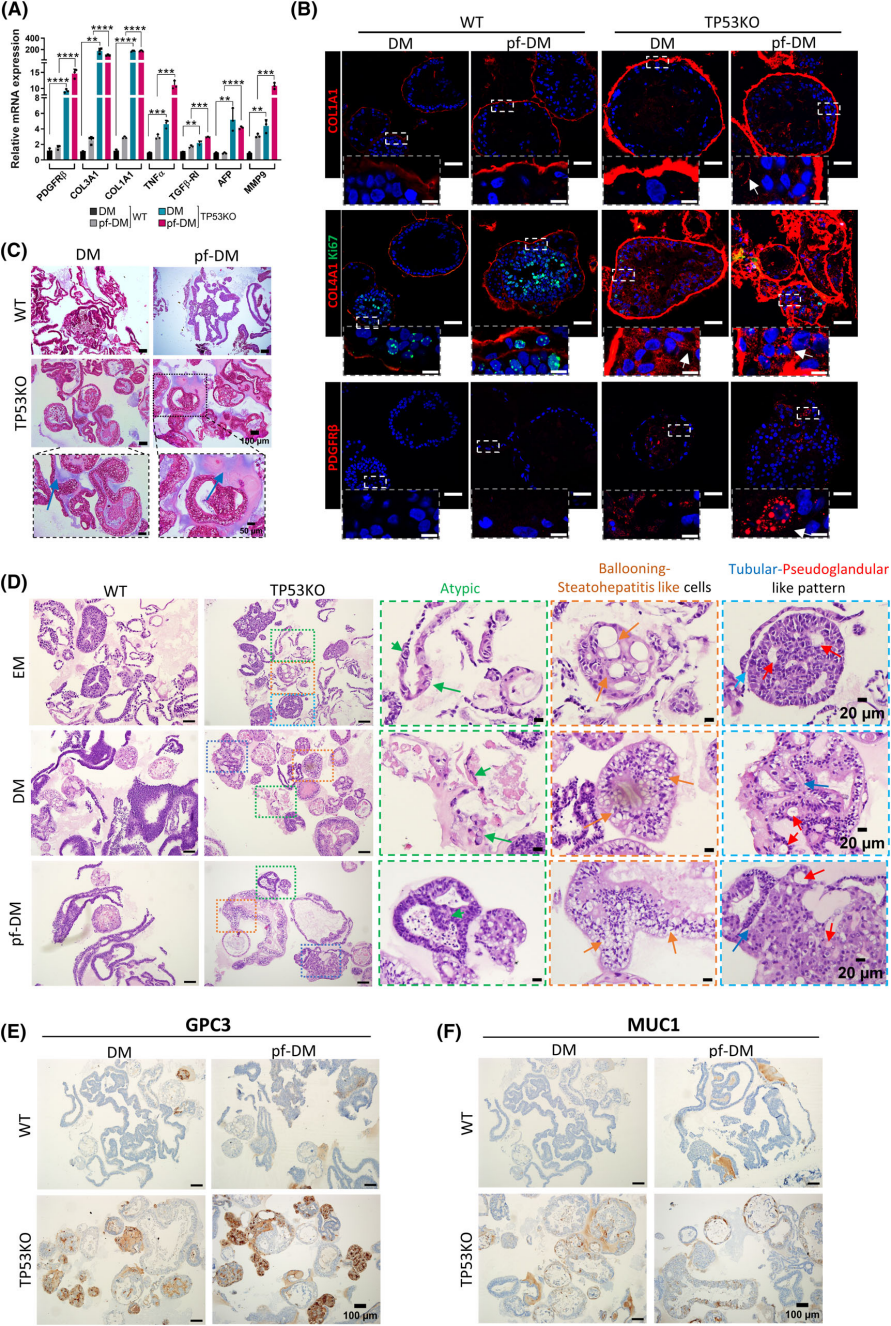
Enhanced fibrotic signature in TP53KO‐eHEPOs. (A) Genes that are significantly overexpressed in TP53KO clones in both DM and pf‐DM conditions are shown in the graph (‘> 0.05’ (n.s.), ‘≤ 0.05’ (*), ‘≤ 0.01’ (**), ‘≤ 0.001’ (***), ‘≤ .0001’ (****), (Student *t*‐test, *n* = 3, SD)). (B) The immunofluorescence (IF) staining of WT and TP53KO‐eHEPOs for fibrosis markers from OCT sections (Scale: 50 μm, Zoom‐in scale: 10 μm), (*n* = 3)). (C) The Masson's trichrome staining in WT and TP53KO‐eHEPOs represent the enrichment level of collagen deposition TP53KO‐eHEPOs in DM and pf‐DM (Scale: 100 μm, Zoom‐in scale: 50 μm, (*n* = 3)). (D) H&E staining of WT and TP53KO‐eHEPOs in EM, DM, and pf‐DM (Scale: 100 μm, Zoom‐in scale: 20 μm, (*n* = 3)). The green arrows show atypical cells, orange arrows show ballooning‐steatohepatitis‐like cells, and blue and red arrows show tubular, pseudo‐glandular rosettes, respectively. (E) IHC staining of glypican‐3 (GPC3) marker represents elevated signal of TP53KO‐eHEPOs (Scale: 100 μm, (*n* = 3)) (F) IHC staining of mucin1 (MUC1) marker represents elevated signal of TP53KO‐eHEPOs (Scale: 100 μm, (*n* = 3)).

To validate our RNA‐seq results, we investigated the expression of established markers of ECM modification and fibrosis. We observed significant upregulation of fibrosis‐associated genes (COL1A1, COL3A1, and MMP9) and key markers of hepatic injury and fibrogenesis (PDGFRB, AFP, TGFB‐R1, and TNF) in both DM and pf‐DM conditions compared to WT‐eHEPOs (Fig. 4A). In addition to this, immunofluorescence staining confirmed the upregulation of COL1A1, COL4A1, and PDGFRB in TP53KO‐eHEPOs compared to WT‐eHEPOs. Notably, IF staining revealed a significant enrichment of PDGFRB, a marker for hepatic stellate cells (HSCs) and fibrosis, in TP53KO‐eHEPOs (Fig. 4B). This suggests that TP53 deficiency in our model may promote the activation of HSCs, which are key players in hepatic fibrosis.

To further validate the increased collagen levels and ECM alterations affecting fibrotic morphology, we conducted Masson's trichrome staining on WT and TP53KO‐eHEPOs. We observed enhanced collagen storage in the TP53KO organoids, and this effect got stronger in the pf‐DM medium (Fig. 4C). Importantly, we conducted COL1A1 and Masson's trichrome staining in different TP53KO‐eHEPO clones (B2 and B8), confirming that COL1A1 expression is slightly elevated, and Masson's staining is significantly higher in B2 and B8 TP53KO‐eHEPO clones compared to the WT (Fig. S6A).

Finally, our investigation revealed that TP53KO‐eHEPOs exhibited altered histopathological changes associated with hepatic fibrosis. These markers included atypical cell morphology (pleomorphic and hyperchromatic multinucleation), pseudo‐glandular‐tubular rosettes, steatohepatitis‐like inflammatory areas, and ballooning‐like hepatocytes enriched in TP53KO‐eHEPOs, particularly in pf‐DM, followed by DM and EM media, respectively, compared to WT‐eHEPOs [32, 33] (Fig. 4D). Similarly, we confirmed the same patterns in other clones, B2 and B8 (Fig. S6B). Altogether, TP53KO‐eHEPOs demonstrated increased fibrosis markers, enhanced collagen deposition, and fibrotic histopathological changes, particularly in pf‐DM conditions, highlighting the augmented fibrotic signature.

### Early disease initiation markers enriched in TP53KO‐eHEPOs

To explore the role of TP53KO‐eHEPOs in liver disease initiation and progression, we conducted IHC staining for well‐known liver disease initiation markers, glypican‐3 (GPC3) and mucin1 (MUC1), in DM and pf‐DM medium. These markers were significantly enriched in our TP53KO‐eHEPOs (Fig. 4E,F). Furthermore, we also investigated the same markers using two other TP53KO clones, B2 and B8. We observed a slight increase in signal in the DM condition. This observation suggests that the absence of TP53 may contribute to the early initiation and progression of liver disease, with this effect being enhanced in pf‐DM (Fig. S6C).

### Hepatic fibrogenesis‐related signaling pathway alterations in TP53KO‐eHEPOs

**Fig. 5:**
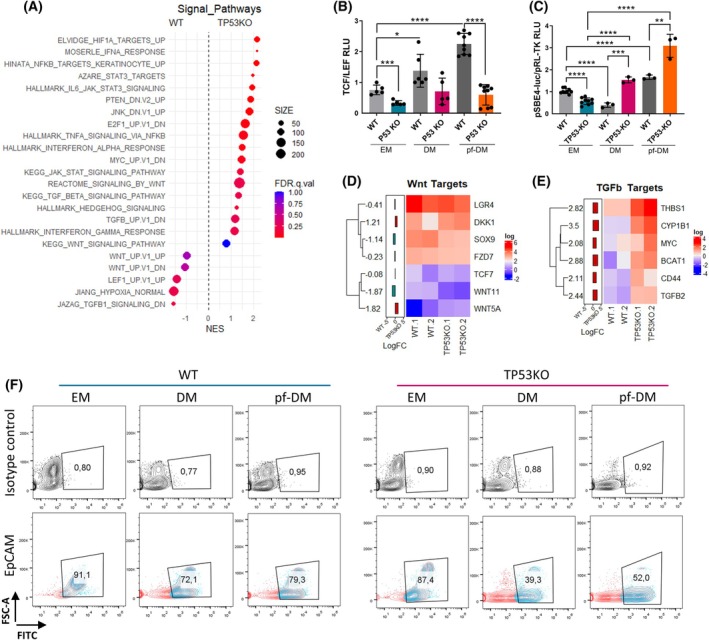
Altered signal pathways in the TP53KO‐eHEPOs. (A) The dot plot of selected signal pathways enrichment score. The figure represents the minus multiplication value of the NES score. The HIF1a, IFNA, STAT3, and TGFB‐related signal pathways significantly enriched in TP53KO‐eHEPOs. (B) The TCL/LEF reporter assay represents attenuated levels of Wnt signal pathways at the transcriptome level. Graphs prepared in graphpad Prism (‘> 0.05’ (n.s.), ‘≤ 0.05’ (*), ‘≤ 0.01’ (**), ‘≤ 0.001’ (***) ‘≤ 0.0001’ (****), (*n* = 5 (EM), *n* = 6 (DM), *n* = 9 (pf‐DM) for TCF\LEF, Student *t*‐test, SD)). (C) The pSBE4‐luc/pRL‐TK reporter assay represents augmented levels of TGFB signal pathways at the transcriptome level. Graphs prepared in the graphpad Prism (‘> 0.05’ (n.s.), ‘≤ 0.05’ (*), ‘≤ 0.01’ (**), ‘≤ 0.001’ (***)‘≤ 0.0001’ (****), (*n* = 9 (EM), *n* = 3 (DM), *n* = 3 (pf‐DM), Student *t*‐test, SD)). (D, E) The heatmap of Wnt and TGFB signal pathways related to enriched genes (log (matrix values), default (logFC), *P* ≤ 0.05). (F) The flow cytometry plots represent the changes in TP53KO‐eHEPOs EpCAM surface marker expressions at EM and day 12 for DM and pf‐DM medium. Plots prepared in FlowJo software (*n* = 3). The percentages display European decimal formatting by default (‘0,80’ refers to ‘0.80’).

To investigate the signaling pathways underlying the observed hepatic fibrogenesis in our model, we conducted a detailed investigation of WT and TP53KO‐eHEPOs. Our observations indicated a significant enrichment in various pathways, including HIF1A‐mediated hypoxia, IL6/STAT3, NFKB, IFN signal‐mediated inflammation, and signals related to MYC. Notably, we also observed an increase in the TGF‐β signal pathway in TP53KO‐eHEPOs, which is a prominent marker of fibrosis. However, the alterations in the Wnt signaling pathway exhibited less pronounced trends and displayed varying patterns among different groups (Fig. 5A). To gain deeper insights into the alterations of these two signaling pathways in our eHEPO model, we examined their transcriptomic levels using a TCF/LEF and pSBE4luc luciferase reporter assay. We noted a significant reduction in the Wnt signaling pathway in TP53KO‐eHEPOs in both DM and pf‐DM medium, while it increased in the WT counterpart (Fig. 5B). As expected, we observed a significant increase in the TGF‐β signaling pathway in TP53KO‐eHEPOs in DM, and this effect was amplified in pf‐DM (Fig. 5C). Additionally, we observed enrichment of Wnt‐related genes LGR4, SOX9, FZD7, and TCF7 and tumor suppressor‐like molecule *WNT11* [34] in the WT‐eHEPOs. However, the Wnt pathway suppressive gene, *DKK1*, and noncanonical *WNT5A*, known for inhibiting the Wnt canonical pathway, were augmented in TP53KO‐eHEPOs (Fig. 5D and Table S3). Importantly, we examined TGF‐β‐related genes, which are drivers for fibrotic transformation and disease progression, such as *TGFB2*, *CD44*, *BCAT1*, and THBS1, and identified significant enrichment in TP53KO‐eHEPOs (Fig. 5E). In summary, we found that TP53KO‐eHEPOs showed enrichment of hepatic fibrosis‐related signal pathways, increased TGF‐β signaling, a key marker of fibrosis, and reduced Wnt signaling compared to WT‐eHEPOs.

### TP53‐mediated regulation of protumorigenic markers in the fibrotic‐eHEPO model

Importantly, TP53 has been reported to directly bind to or have a close relationship with protumorigenic cell markers and their promoter regions to regulate their expression [35, 36]. To understand their possible regulations and contributions to liver fibrosis initiation and progression, we checked their expression patterns. There, we observed a significant decrease in the number of EpCAM‐positive cells in TP53KO‐eHEPOs, consistent with our transcriptome assay (Fig. 5F). Similarly, we noted a reduced number of CD24‐positive cells in TP53KO‐eHEPOs in the DM medium. However, there was an increased expression of CD133‐positive cells in the DM medium in TP53KO‐eHEPOs. Interestingly, the pf‐DM medium, regardless of TP53 status, increased the proportion of positive cells. Finally, we did not observe any clear changes in CD44 and CD90 markers (Fig. S7A). Following these observations, TP53KO‐eHEPOs showed a decrease in EpCAM and CD24‐positive cells, but an increase in CD133‐positive cells under DM medium. The pf‐DM medium increased positive cell proportions regardless of TP53 status, with no changes in CD44 and CD90 markers.

### DNA damage and distribution significantly increase in TP53KO‐eHEPOs

Genomic integrity and maintenance represent another major role of the *TP53* gene. When cellular DNA damage occurs, it is recognized by the DNA damage response (DDR) pathways composed of sensors, transducers, and effectors. For example, in the case of double‐strand DNA breaks, the ataxia telangiectasia mutated (ATM) molecule is recruited and activated, facilitating the phosphorylation of the histone variant H2AX at the S139 residue. Subsequently, H2AX recruits DNA damage checkpoint protein 1 (MDC1), which mediates the recruitment of several proteins to the damaged DNA site, including TP53‐binding protein 1 (TP53BP1). This leads to the binding and phosphorylation of TP53, activating and controlling cell cycle arrest and apoptosis [37]. In our WT and TP53KO‐eHEPOs, we also assessed the amount of DNA damage mediated by H2A.X Ser139 in DM and pf‐DM. We observed that TP53KO‐eHEPOs accumulated DNA damage in DM, and the pf‐DM medium augmented this effect. Additionally, we observed a slight increase in cleaved caspase‐3 in DM. However, the pf‐DM medium stimulates more cell death and possible hepatic injury in both WT and TP53KO‐eHEPOs (Fig. S7B). As a result, TP53KO‐eHEPOs exhibit increased DNA damage due to the loss of P53‐mediated elimination of damaged cells.

## Discussion

Several studies have demonstrated that TP53 is a key regulator of apoptosis, senescence, cell cycle, fibroblast activation, and ECM production, indicating its role in fibrosis initiation and progression. However, most of these studies are limited by 2D experimental cell‐culture systems or nonphysiologically relevant mouse models [38]. The lack of reliable human models that mimic precancerous fibrogenesis in the early stage of the disease remains a significant obstacle. In this study, we developed a CRISPR/Cas9‐mediated TP53KO human eHEPO model to mimic hepatic fibrogenesis within different microenvironments.

The functional loss of TP53 results in aberrant regulation of cell death, proliferation, and differentiation, a characteristic observed in the majority of cancers [38, 39]. As expected, we observed that our TP53KO‐eHEPOs exhibit increased proliferation in the EM medium. However, TP53KO‐eHEPOs exhibited reduced proliferation in DM and pf‐DM media compared to WT. This may be due to the loss of TP53's role in genomic barrier/stability function; nonetheless, our differentiation‐specific media (DM and pf‐DM) effectively push the organoids toward differentiation. In this regard, we conducted multiple analyses to further assess the differentiation state of our TP53KO‐eHEPOs. As expected, the results indicated well‐differentiated hepatic cell characteristics in the TP53KO‐eHEPOS, including increased ALB secretion, positive PAS staining (glycogen storage), and enhanced A1AT, ALB, and CK18 staining. Importantly, our model shares certain similarities with findings in a TP53 knockout mouse model, where knockout cells exhibited a blast‐like shape and increased albumin secretion, characterized by hyperproliferative behavior and the retention of protumorigenic properties [40].

To explore the transcriptomic changes associated with TP53 and its potential effect on the fibrotic transformation of those hepatocytes, we performed RNA‐seq analysis on WT and TP53KO organoids in DM conditions. Genome‐wide transcriptome and comparative GSEA analysis in TP53KO‐eHEPOs revealed enrichment in gene sets related to fibrosis, EMT, ECM modification, and inflammation. These findings suggested a potential role of TP53 in regulating the fibrotic transformation process. Consistent with these transcriptomic findings, we further investigated these processes at the protein and histological levels. We performed qPCR and IF staining with well‐known markers. Importantly, we observed that ECM modification and liver fibrosis‐related marker gene expressions were significantly upregulated in TP53KO‐eHEPOs, including *COL3A1*, *COL1A1*, *TGFB‐R1*, *TNFa*, *AFP*, and *MMP9*. More importantly, activated hepatic stellate cell marker, PDGFRB, gene expression, and staining were also significantly upregulated in TP53KO organoids, providing evidence of hepatic stellate cell occurrence and hepatic fibrogenesis in our model. In addition, we further confirmed hepatic fibrogenesis with Masson's trichrome staining and associated histological changes, including heightened atypical features, pseudo‐glandular‐tubular rosettes, steatohepatitis‐like inflammatory areas, and ballooning‐like hepatocytes, accompanied by increased collagen deposition. Similar histological changes and an elevated collagen signal have also been reported in various studies [3, 33, 41, 42]. For example, one study demonstrated that thioacetamide (TAA) and free fatty acids (FFA) induced hepatic organoids to increase *COL1A1*, *COL3A1*, and *PDGFRB* gene expressions, as well as alterations in the ECM modification and pro‐inflammatory cytokines, resembling fibrosis, and steatosis‐like histological changes. In addition, a pioneering study demonstrated that iPSC‐derived organoid models can be induced to develop steatohepatitis‐like pathology and fibrosis through free fatty acid treatment [43]. While this work provides strong evidence for fibrosis in organoid systems, it primarily focuses on fibrosis resulting from steatohepatitis. In contrast, our model simulates a fibrotic microenvironment driven by the loss of function of the tumor suppressor gene TP53 (via knockout), reflecting a more physiological route to fibrosis. This is further supported by the presence of activated stellate cells and macrophages. In summary, although both studies investigate fibrosis, our model specifically represents TP53KO‐mediated fibrosis, whereas the other model captures fibrosis associated with steatohepatitis.

Importantly, similar to our pf‐DM‐treated TP53KO‐eHEPO results, in this study authors showed that collagen staining became thick around the perisinusoidal space after treatment [42]. This phenomenon is related to the mechanical plasticity of the organoids. According to the literature, an interesting study demonstrated that the collective back‐and‐forth motion of cells within the branches of mammary gland organoids generates tension strong enough to induce a plastic reorganization of the surrounding collagen network. This reorganization results in the formation of mechanically stable collagen cages that encase the growing organoid branches [44]. Importantly, Fig. 4B shows that WT‐eHEPOs exhibit limited mechanical deformation (accumulation), whereas TP53KO‐eHEPOs undergo significant deformation, which correlates with their increased migration capacity within the fibrotic microenvironment [45, 46, 47]. Additionally, we observed that the central zone exhibits augmented positive PDGFRB and COL4A1 staining in TP53KO‐eHEPOs, compared to WT. In addition to PDGFRB staining, we also confirm the presence of stellate cells in our organoid model with a‐SMA staining, as shown in Fig. S1A. Stellate cells are generally located on the inner stromal side of the organoid, with some also positioned near the corners. We believe the location of aSMA‐positive cells correlates with PDGFRB and COL4A1 staining. Together, these data support the conclusion that activated hepatic stellate cells are the most likely source of accumulated collagen in TP53KO‐eHEPOs. Additionally, we confirm that our TP53KO‐eHEPO model represents the enriched expression of well‐known liver disease initiation markers, glypican‐3 (GPC3) and mucin1 (MUC1), compared to WT, which may contribute to TP53‐mediated disease progression [48, 49, 50, 51].

Our transcriptome analysis revealed enrichment in signaling pathways associated with TP53 function, including *HIF1α*, *IFNα*, and *STAT3*. Interestingly, a recent study suggests that IL10‐mediated senescence in HSCs can activate *TP53* via *STAT3* signaling, leading to reduced fibrosis [52]. However, in our TP53KO model, the lack of *TP53*, potentially caused by aberrant activation of downstream STAT3 signals, might contribute to the observed fibrotic phenotype. This aligns with findings in other cancers, where TP53 deficiency is associated with poor prognosis [52, 53].

We also observed an upregulation of the TGFβ signaling pathway and a downregulation of the Wnt signaling pathway, along with an increase in Wnt inhibitory signals such as *DKK1* and *CDKN2A*, in TP53KO‐eHEPOs. A recent study linked fibrous nest‐type HCCs with Hoshida's liver subset S1, characterized by alterations in Wnt/TGFβ signaling, inflammation, and ECM modification, and associated with poor patient outcomes [54]. Interestingly, our TP53KO‐eHEPOs also exhibited enrichment of S1, suggesting a similar underlying mechanism. This observed correlation further validates our eHEPO model as a valuable tool for investigating the progression of fibrosis.

To provide insights into the clinical significance of the enriched signal pathway‐related genes within our DEG list, we conducted the Kaplan–Meier plotter analysis. The individuals with elevated expression levels of *COL24A1, S100A9*, and *COL11A1* (Fig. S8A), *CXCL3*, *CXCL5*, and *CCL20* (Fig. S8B), and *ADAM12*, *SPP1*, and *ITGA2* (Fig. S8C), along with *KRT80*, *MUC15*, and *MUC1* (Fig. S8D), exhibited lower overall survival rates. These findings underscore the potential prognostic significance of these genes in the early stage of hepatic fibrogenesis.

Additionally, we examined alterations in protumorigenic cell markers in our eHEPO model due to their direct or indirect regulation by *TP53*. EpCAM is generally highly expressed in the early developmental stages of the liver, including embryonic stem cells, endoderm, liver progenitors, and immature hepatocytes. Its expression is attenuated during hepatocyte differentiation; however, EpCAM expression level increases once more when cells enter progenitor or cancer‐like stages [55]. Consistent with this, we observed a significant reduction in EpCAM‐ and CD24‐positive cells in TP53KO‐eHEPOs cultured in DM and pf‐DM (well‐differentiated stages) compared to EM (hepatic progenitors) conditions [56]. However, we observed an increase in CD133 in TP53KO‐eHEPOs in DM and pf‐DM conditions, which may facilitate an aggressive inflammatory and fibrotic response [57, 58]. This finding aligns with a recent study showing increased CD44 and CD133 levels in patient‐derived TP53KO organoids, indicating protumorigenic properties [59]. Furthermore, we confirm that DNA damage and distribution significantly increase in TP53KO‐eHEPOs parallel with histological and fibrotic alterations.

## Conclusions

In conclusion, our *in vitro* eHEPO model demonstrated a more realistic representation of human physiology and gene expression patterns, aligning closely with the current knowledge in the literature regarding liver fibrogenesis. It offers a valuable tool for studying the effects of TP53‐like first‐hit genes on hepatic progenitor cell transformation in various environments. We anticipate this model's utility in investigating the combined effects of microenvironments and gene knockouts, making it a promising platform for early stage drug discovery.

## Conflict of interest

The authors declare no conflict of interest.

## Author contributions

MK performed all *in vitro* experiments, analyzed data, prepared the figures, and wrote the paper. LB and GK conducted the initial bioinformatic analysis. MK visualized the bioinformatic analysis results. CC and CC contributed to the CRISPR/Cas9 experiments. OO performed pathological evaluations of WT and TP53KO‐eHEPOs. SS designed the CRISPR knockout of p53 and provided experimental guidance. SA discussed the project, performed revision‐related *in vitro* experiments, critically reviewed, and edited the paper. EE conceived and conducted the study, analyzed data, and finalized the paper. The paper has been reviewed and approved by all named authors.

## Supporting information


**Fig. S1.** iPSC‐derived‐eHEPO model faithfully represents mesenchymal origin (SMA and CD68 positive) cells. (A) The a‐SMA staining of WT and TP53KO eHEPOs within DM and pf‐DM mediums (*n* = 3), (Scale: 20 μm). Images were taken with DIC microscope (Olympus IX71). IHC staining of a‐SMA represents hepatic stellate cells located around membrane and lumen of eHEPOs (Scale: 100 μm). (B) CD68 staining of WT and TP53KO eHEPOs within DM and pf‐DM mediums (*n* = 3) (Scales: 20 μm and 5 μm (in zoom)). Images were taken with DIC microscope (Olympus IX71). (C) Schematic outlining of gRNA and target location on TP53 (top). Representative Sanger DNA sequencing revealed that the selected clones had successfully evolved a frameshift mutation in the desired location (bottom). (D) Transfection efficiency after gRNA transformation to the WT‐eHEPOs (*n* = 7) (Scales: 500 and 50 μm). Images were taken with a DIC microscope (Olympus IX71) Green: GFP.

**Fig. S2.** pf‐DM production and validation. (A) The schematic showing of the production of activated LX‐2 stellate cell secretome (left). LX‐2 cells before and after induction TGFB (10 μm, 24 h) (middle), and confirmation with qPCR markers specific for activated LX‐2 stellar cells (right). (B) The schematic showing the production M2 polarized macrophage cell secretome (top). The morphology of the THP1, MO and M2 cells before and after induction (bottom‐left), and confirmation with qPCR markers specific for M2 macrophages (bottom‐right). (C) The validation of p53 gRNA in the HEK293T cell line. Transfected HEK293‐T cells (2.5 μg plasmid at a 1 : 5 μg DNA/PEI ratio for 6 h) collected at day 6 and 50 ug protein loaded for bulk analysis. C1 and C2 represent a clonal growth of selected single cells from several single‐cell clones previously obtained and cultured. Calnexin used as loading control. (1 : 1000).

**Fig S3.** CRISPR/Cas9‐mediated TP53KO‐eHEPOs. (A) The flow cytometry results represent GFP positive cells percentage 24 h later of the gRNA transfection (top). Cell viability was assessed by counting DAPI‐negative cells (bottom). (B) The schematic representation of sanger sequence results for the other growing clones, B2, B5, B6, B8, and D2. The arrow indicates Adenine nucleotide insertion followed by PAM sequence. (C) IHC staining of H&E, Ki67 and P53 in the other clones, B2, B5, B6, B8, and D2.

**Fig S4.** The proliferation and differentiation status of TP53KO‐eHEPOs. (A) Ki67‐positive cells in WT and TP53KO‐eHEPOs within DM and pf‐DM mediums. (B) IHC staining of WT and B2, B8 TP53KO‐eHEPO clones also represent enrichment of differentiation markers A1AT, ALB and PAS staining.

**Fig S5.** Transcriptome of eHEPOs. (A) Light microscope images of eHEPOs prior to RNAseq. (B, C) The dot plot of selected WT or TP53KO‐eHEPO enriched signal pathways. The figure represents the minus multiplication value of the NES score. (D) The heatmap of Yamashita's EpCAM upregulated liver cancer subclass (log (matrix values)), default (logFC), *P* ≤ 0.05. (E) The dot plot of selected metabolism related enriched signal pathways. The figure represents the minus multiplication value of the NES score.

**Fig S6.** Fibrotic signature in TP53KO clones. (A) Images represent COL1A1 and Masson's trichrome stain in WT and p53KO clones. (B) H&E staining in WT and p53KO clones. (C) Early disease initiation markers GPC3 and MUC1 stain in WT and p53KO clones. All figures represent similar scale size with the main figure and 10× zoom.

**Fig S7.** Stem cell and DNA damage in TP53KO‐eHEPOs. (A) The flow cytometry of the CD44, CD133, CD24 and CD90 surface markers at EM, DM, and pf‐DM. Plots prepared in Flowjo software (*n* = 3). (B) The IF staining of DNA damage, H2AX‐ser139, and cell death, cleaved‐caspase3, markers in WT and TP53KO‐eHEPOs (Scales: 50 μm).

**Fig S8.** Kaplan Meier Plots of TP53KO‐eHEPOs. (A) The overall survival (OS) Kaplan‐Meier plots of the selected genes related with Fibrosis. (B) Inflammation **C** ECM modification and (D) Tumorigenesis gene set enriched in the TP53KO‐eHEPOs. All plots employing median and auto‐select best cutoff parameters [1]. Blue frame around COL1A1 represents a non‐significant P value.

**Table S1.** The list of qPCR primers.

**Table S2.** The antibody list.

**Table S3.** (A) Differentially expressed genes (DEG). (B) Upregulated genes (C) Downregulated genes.

**Table S4.** (A) REACTOME_WT (B) REACTOME_TP53KO(B4) (C) ONCOGENE_WT (D) ONCOGENE_TP53KO(B4) (E) KEGG_WT (F) KEGG_TP53KO(B4) (G) HALLMARK_WT (H). HALLMARK_TP53KO(B4) (I) GOBP_WT (J) GOBP_TP53KO(B4) (K) GOCC_WT (L) GOCC_TP53KO(B4) (M) GOMF_WT (N) GOMF_TP53KO(B4) (O) HOSHIDA_CHIANG_WT (P) HOSHIDA_CHIANG_TP53KO(B4).

## Data Availability

The RNA sequencing data generated in this paper is available in NCBI‐Gene Expression Omnibus (GEO) database under the accession number GSE252464 and in SRA datasets under BioProject PRJNA1060695.
