# Supplementary figures and images for: Modeling hepatic fibrosis in TP53 knockout iPSC‐derived human liver organoids
Source: Mol Oncol. 2025 Oct 9;20(3):668–87. doi: 10.1002/1878-0261.70119 (PMC13042547; doi:10.1002/1878-0261.70119)

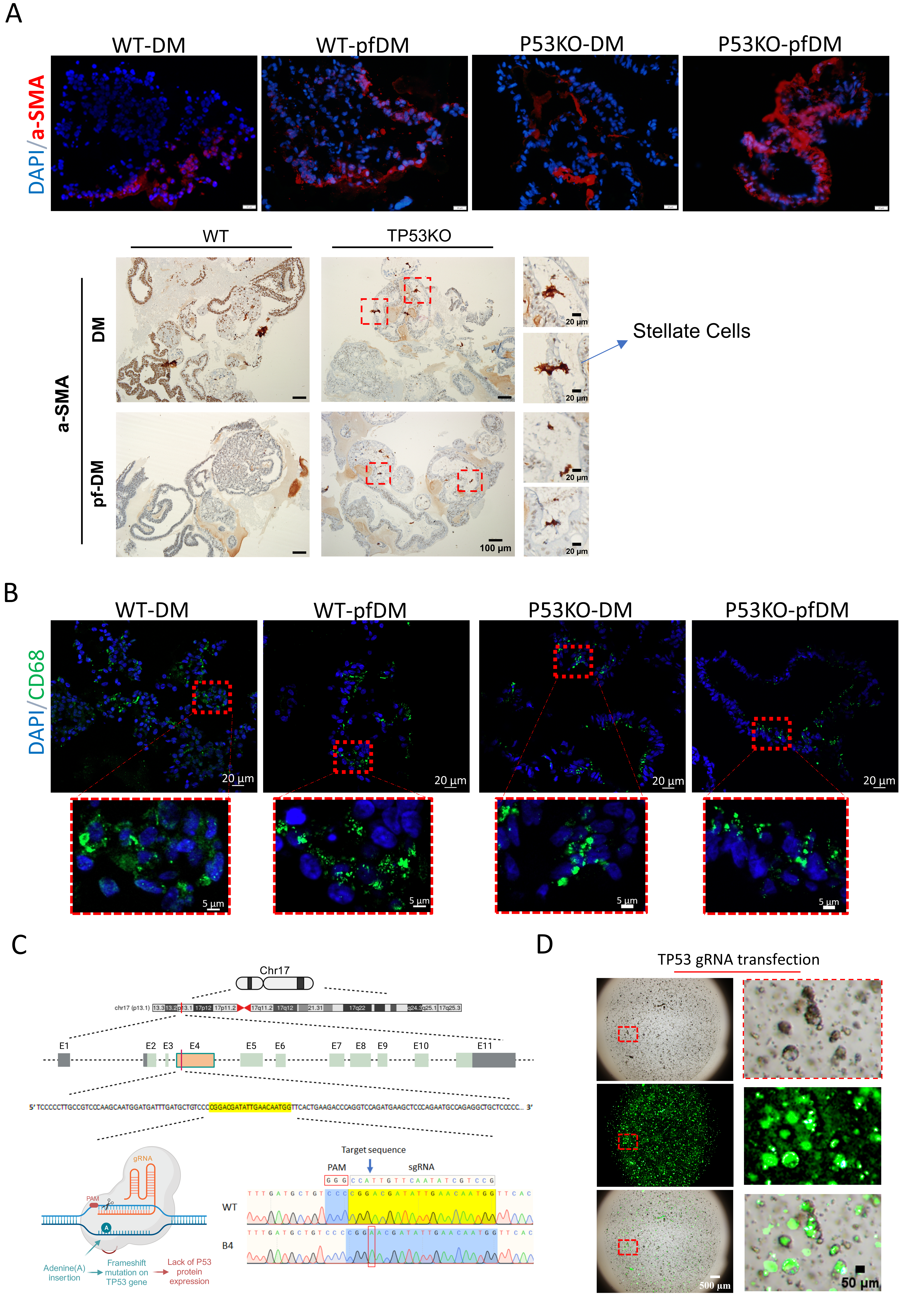

Supplement: Supplementary file 1 — Fig. S1. iPSC‐derived‐eHEPO model faithfully represents mesenchymal origin (SMA and CD68 positive) cells. (A) The a‐SMA staining of WT and TP53KO eHEPOs within DM and pf‐DM mediums (n = 3), (Scale: 20 μm). Images were taken with DIC microscope (Olympus IX71). IHC staining of a‐SMA represents hepatic stellate cells located around membrane and lumen of eHEPOs (Scale: 100 μm). (B) CD68 staining of WT and TP53KO eHEPOs within DM and pf‐DM mediums (n = 3) (Scales: 20 μm and 5 μm (in zoom)). Images were taken with DIC microscope (Olympus IX71). (C) Schematic outlining of gRNA and target location on TP53 (top). Representative Sanger DNA sequencing revealed that the selected clones had successfully evolved a frameshift mutation in the desired location (bottom). (D) Transfection efficiency after gRNA transformation to the WT‐eHEPOs (n = 7) (Scales: 500 and 50 μm). Images were taken with a DIC microscope (Olympus IX71) Green: GFP. [file MOL2-20-668-s004.tif]

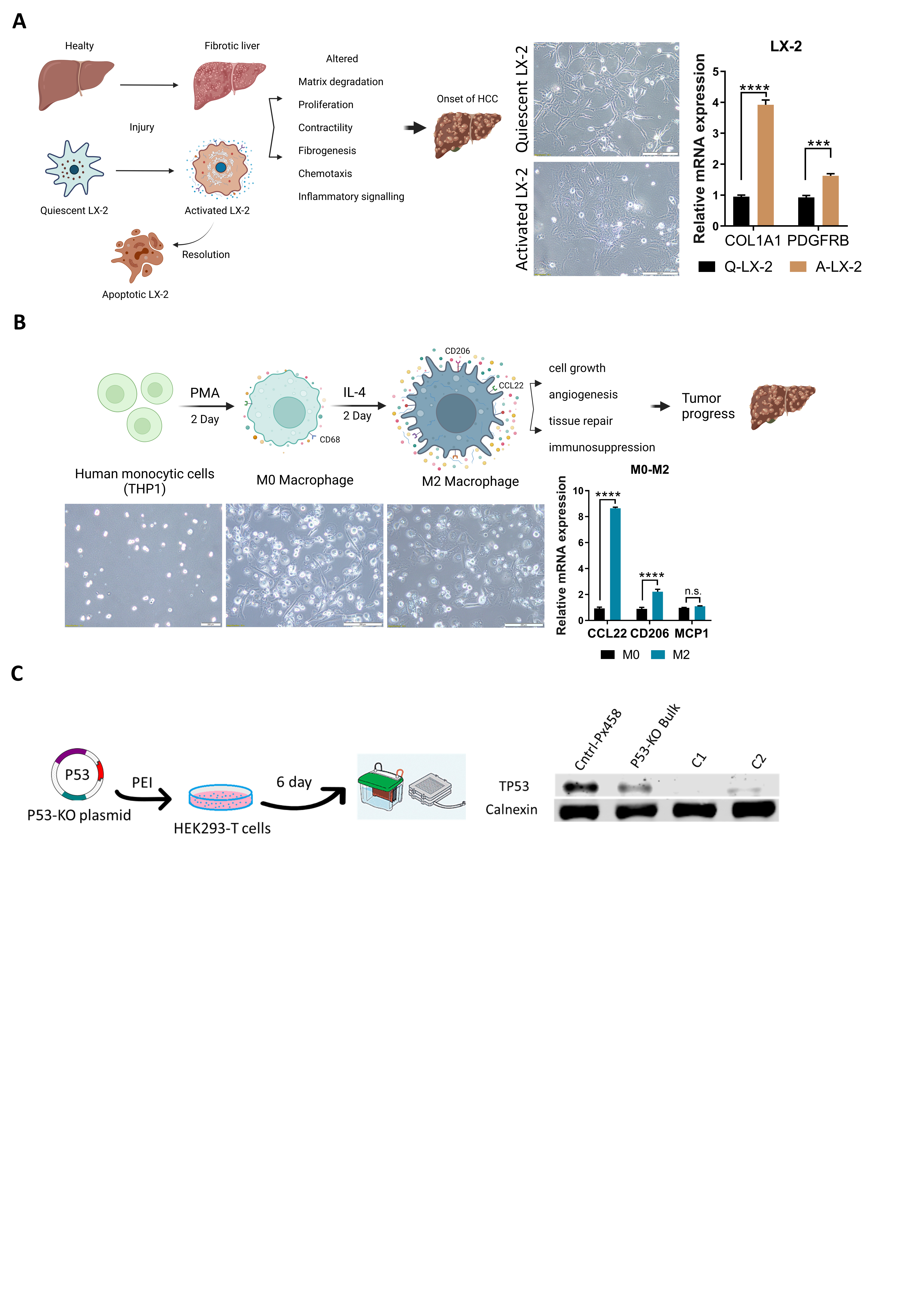

Supplement: Supplementary file 2 — Fig. S2. pf‐DM production and validation. (A) The schematic showing of the production of activated LX‐2 stellate cell secretome (left). LX‐2 cells before and after induction TGFB (10 μm, 24 h) (middle), and confirmation with qPCR markers specific for activated LX‐2 stellar cells (right). (B) The schematic showing the production M2 polarized macrophage cell secretome (top). The morphology of the THP1, MO and M2 cells before and after induction (bottom‐left), and confirmation with qPCR markers specific for M2 macrophages (bottom‐right). (C) The validation of p53 gRNA in the HEK293T cell line. Transfected HEK293‐T cells (2.5 μg plasmid at a 1 : 5 μg DNA/PEI ratio for 6 h) collected at day 6 and 50 ug protein loaded for bulk analysis. C1 and C2 represent a clonal growth of selected single cells from several single‐cell clones previously obtained and cultured. Calnexin used as loading control. (1 : 1000). [file MOL2-20-668-s006.tif]

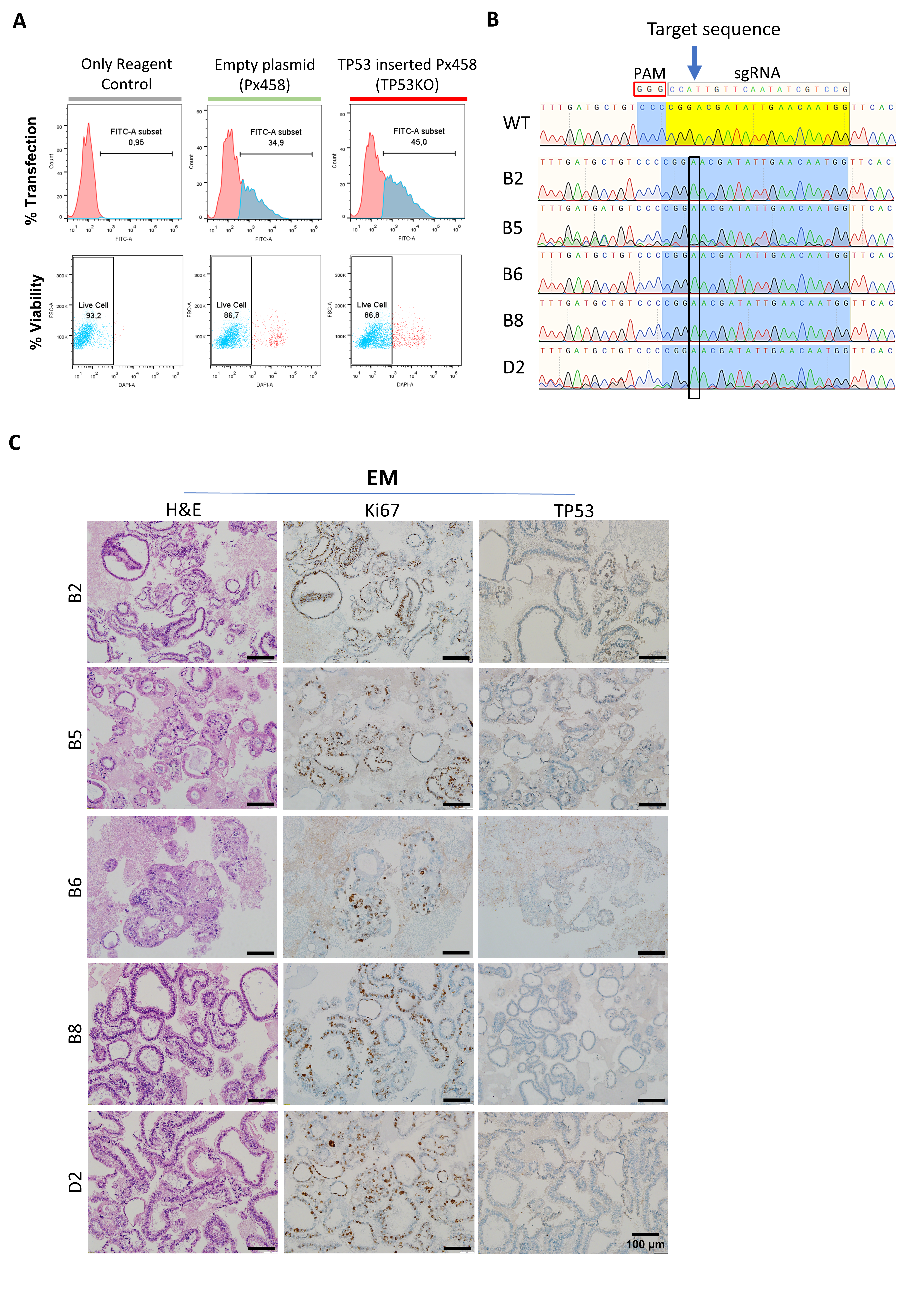

Supplement: Supplementary file 3 — Fig S3. CRISPR/Cas9‐mediated TP53KO‐eHEPOs. (A) The flow cytometry results represent GFP positive cells percentage 24 h later of the gRNA transfection (top). Cell viability was assessed by counting DAPI‐negative cells (bottom). (B) The schematic representation of sanger sequence results for the other growing clones, B2, B5, B6, B8, and D2. The arrow indicates Adenine nucleotide insertion followed by PAM sequence. (C) IHC staining of H&E, Ki67 and P53 in the other clones, B2, B5, B6, B8, and D2. [file MOL2-20-668-s009.tif]

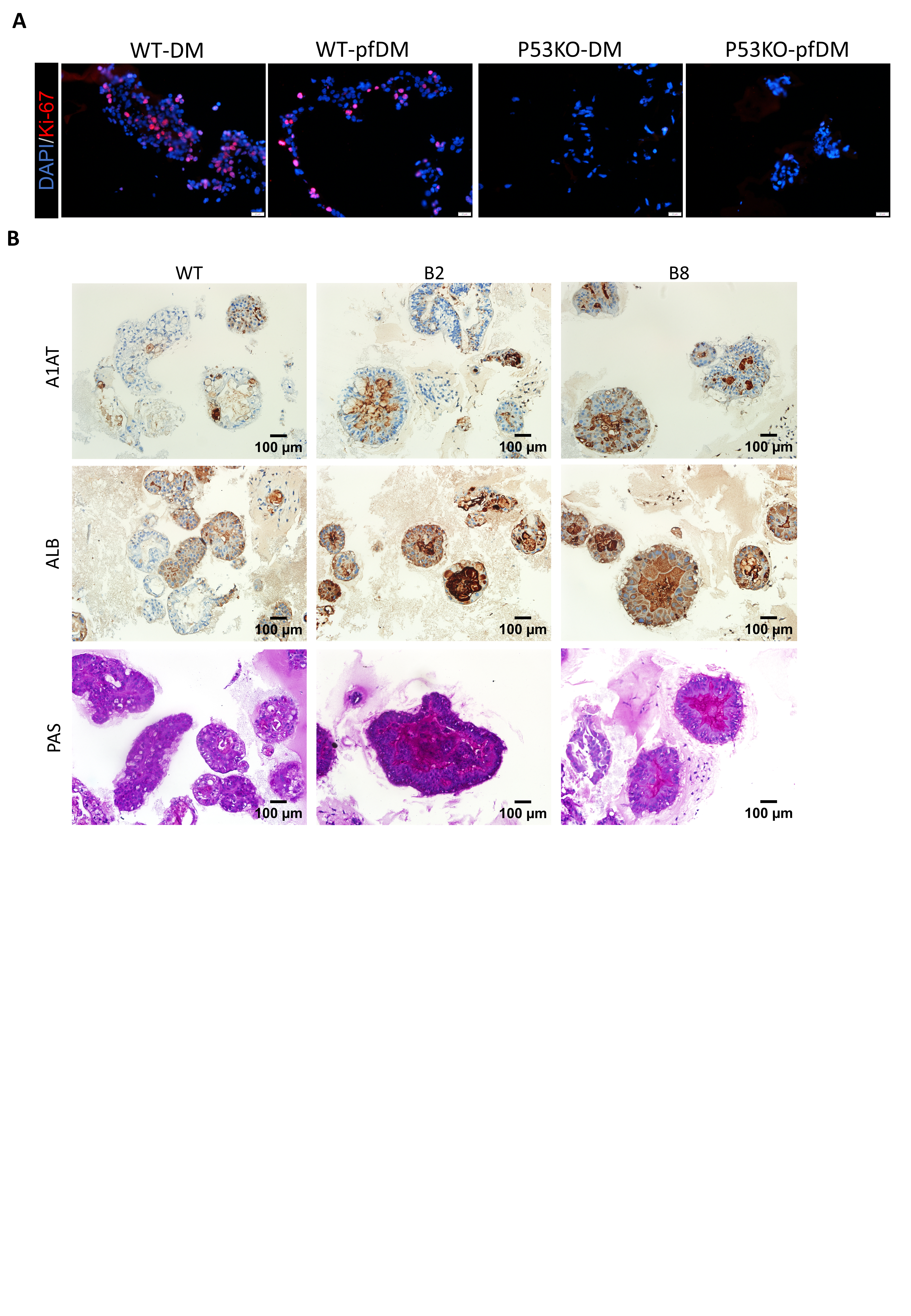

Supplement: Supplementary file 4 — Fig S4. The proliferation and differentiation status of TP53KO‐eHEPOs. (A) Ki67‐positive cells in WT and TP53KO‐eHEPOs within DM and pf‐DM mediums. (B) IHC staining of WT and B2, B8 TP53KO‐eHEPO clones also represent enrichment of differentiation markers A1AT, ALB and PAS staining. [file MOL2-20-668-s007.tif]

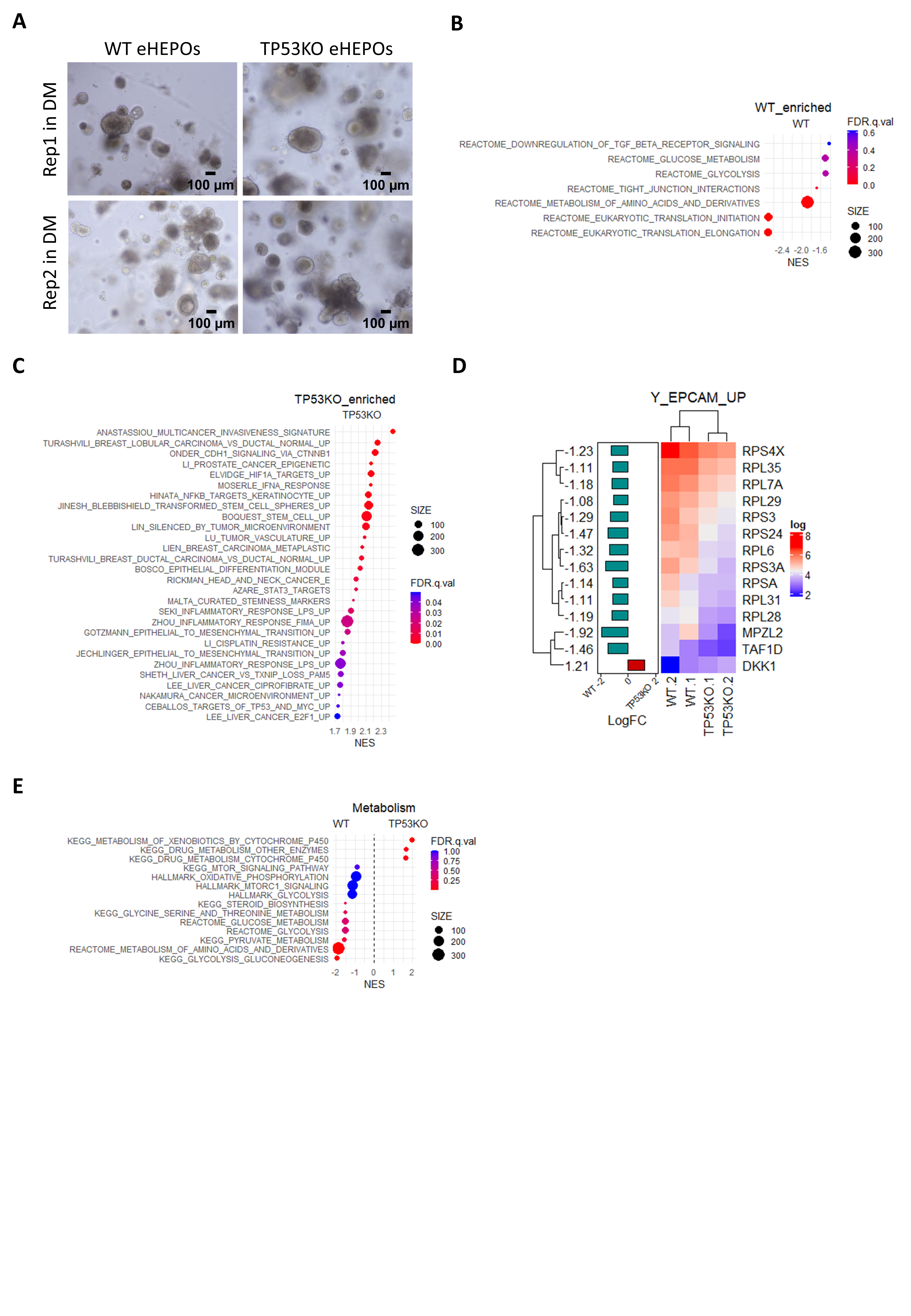

Supplement: Supplementary file 5 — Fig S5. Transcriptome of eHEPOs. (A) Light microscope images of eHEPOs prior to RNAseq. (B, C) The dot plot of selected WT or TP53KO‐eHEPO enriched signal pathways. The figure represents the minus multiplication value of the NES score. (D) The heatmap of Yamashita's EpCAM upregulated liver cancer subclass (log (matrix values)), default (logFC), P ≤ 0.05. (E) The dot plot of selected metabolism related enriched signal pathways. The figure represents the minus multiplication value of the NES score. [file MOL2-20-668-s003.tif]

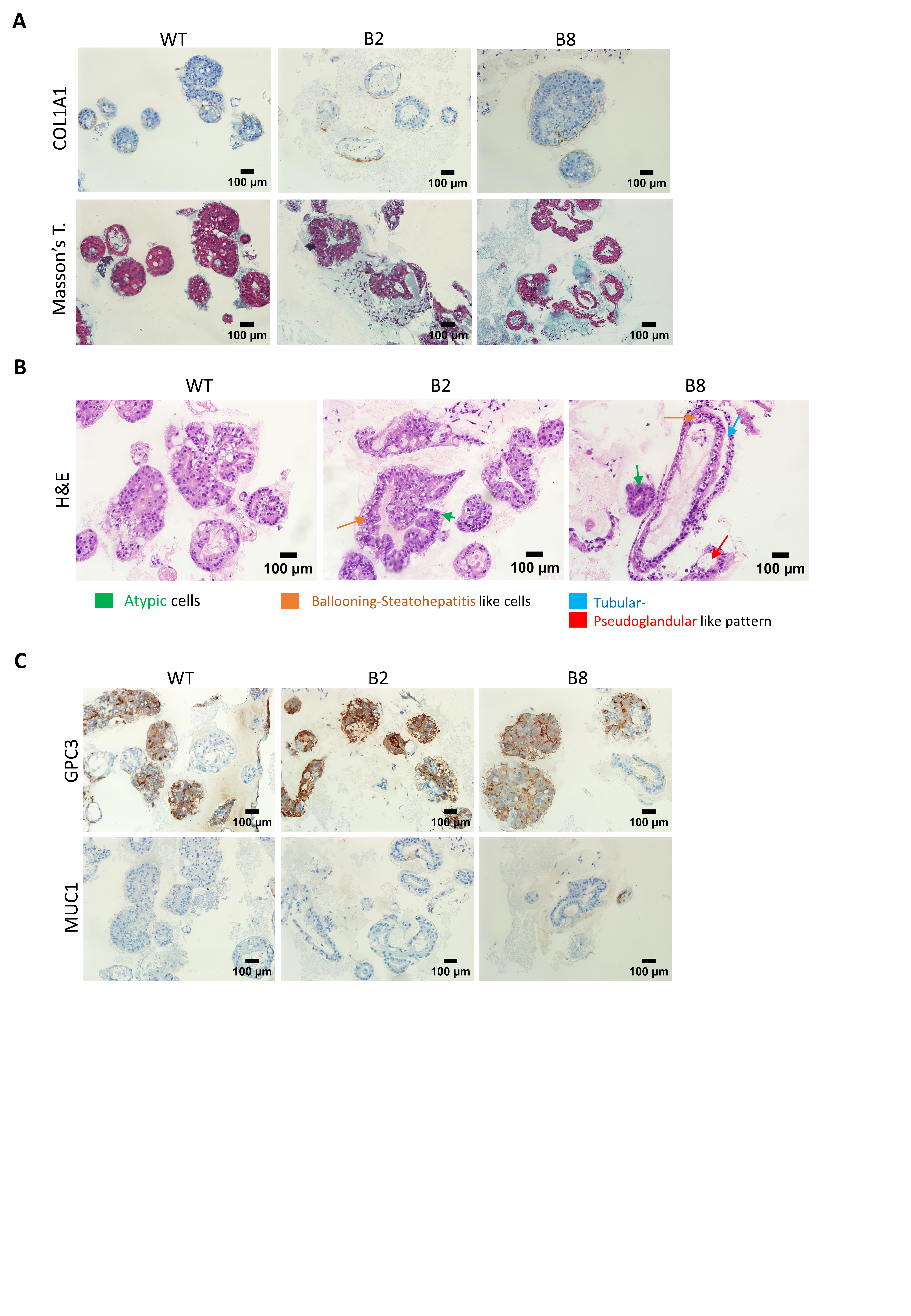

Supplement: Supplementary file 6 — Fig S6. Fibrotic signature in TP53KO clones. (A) Images represent COL1A1 and Masson's trichrome stain in WT and p53KO clones. (B) H&E staining in WT and p53KO clones. (C) Early disease initiation markers GPC3 and MUC1 stain in WT and p53KO clones. All figures represent similar scale size with the main figure and 10× zoom. [file MOL2-20-668-s002.tif]

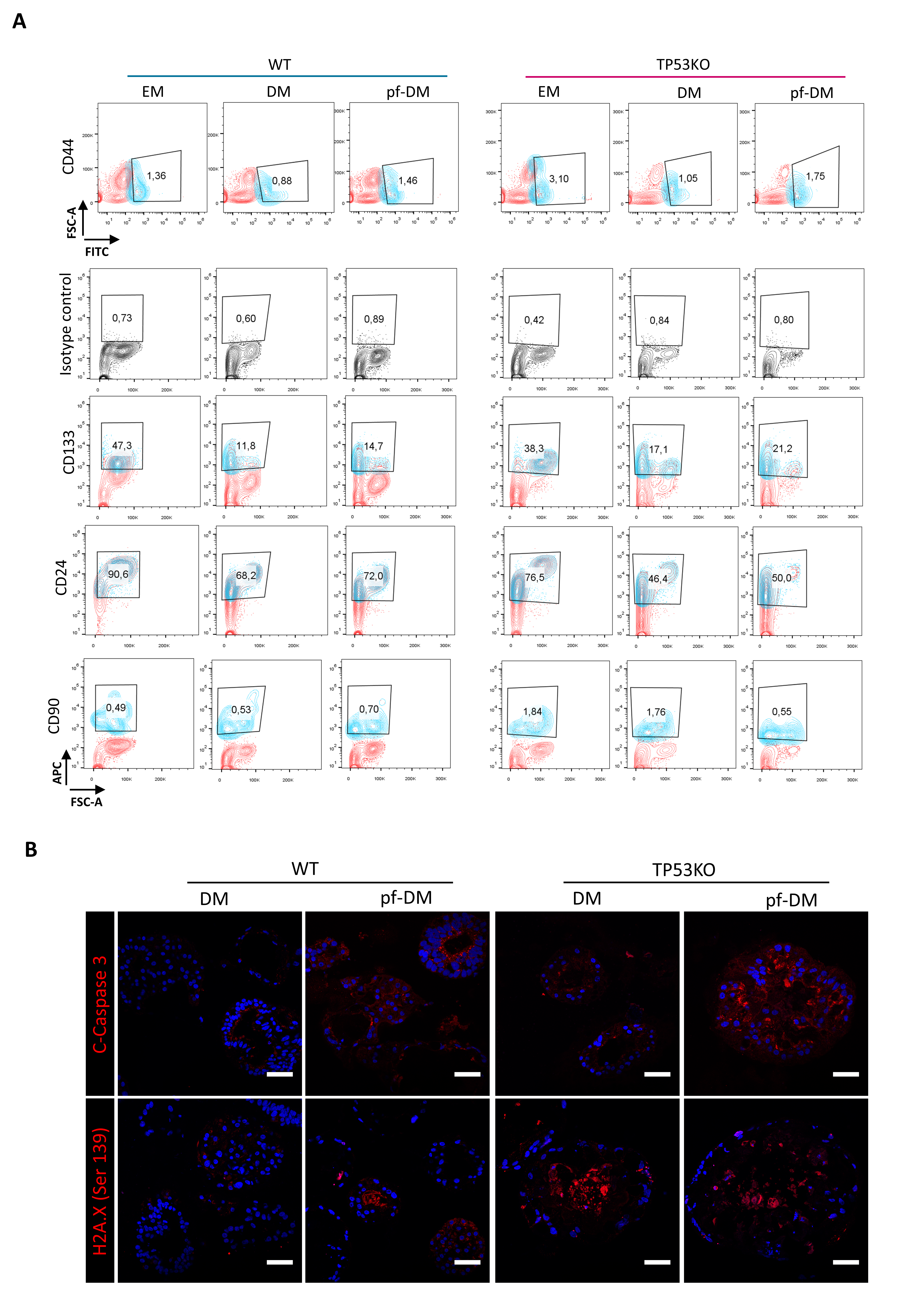

Supplement: Supplementary file 7 — Fig S7. Stem cell and DNA damage in TP53KO‐eHEPOs. (A) The flow cytometry of the CD44, CD133, CD24 and CD90 surface markers at EM, DM, and pf‐DM. Plots prepared in Flowjo software (n = 3). (B) The IF staining of DNA damage, H2AX‐ser139, and cell death, cleaved‐caspase3, markers in WT and TP53KO‐eHEPOs (Scales: 50 μm). [file MOL2-20-668-s001.tif]

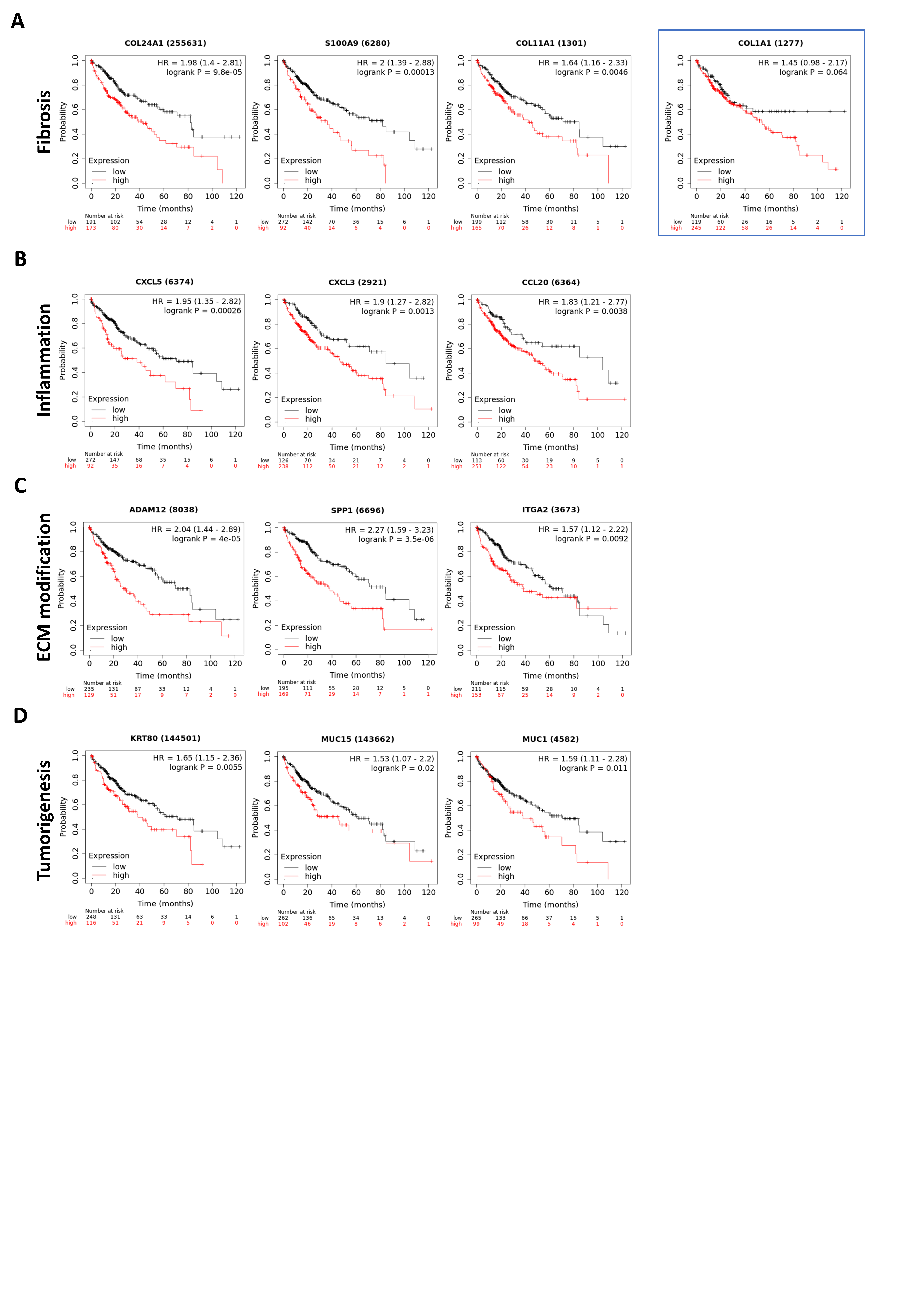

Supplement: Supplementary file 8 — Fig S8. Kaplan Meier Plots of TP53KO‐eHEPOs. (A) The overall survival (OS) Kaplan‐Meier plots of the selected genes related with Fibrosis. (B) Inflammation C ECM modification and (D) Tumorigenesis gene set enriched in the TP53KO‐eHEPOs. All plots employing median and auto‐select best cutoff parameters [1]. Blue frame around COL1A1 represents a non‐significant P value. [file MOL2-20-668-s010.tif]
